# Supplementary figures and images for: Immunosuppression variably impacts outcomes for patients hospitalized with COVID-19: A retrospective cohort study
Source: PLoS One. 2025 Aug 8;20(8):e0330110. doi: 10.1371/journal.pone.0330110 (PMC12334029; doi:10.1371/journal.pone.0330110)

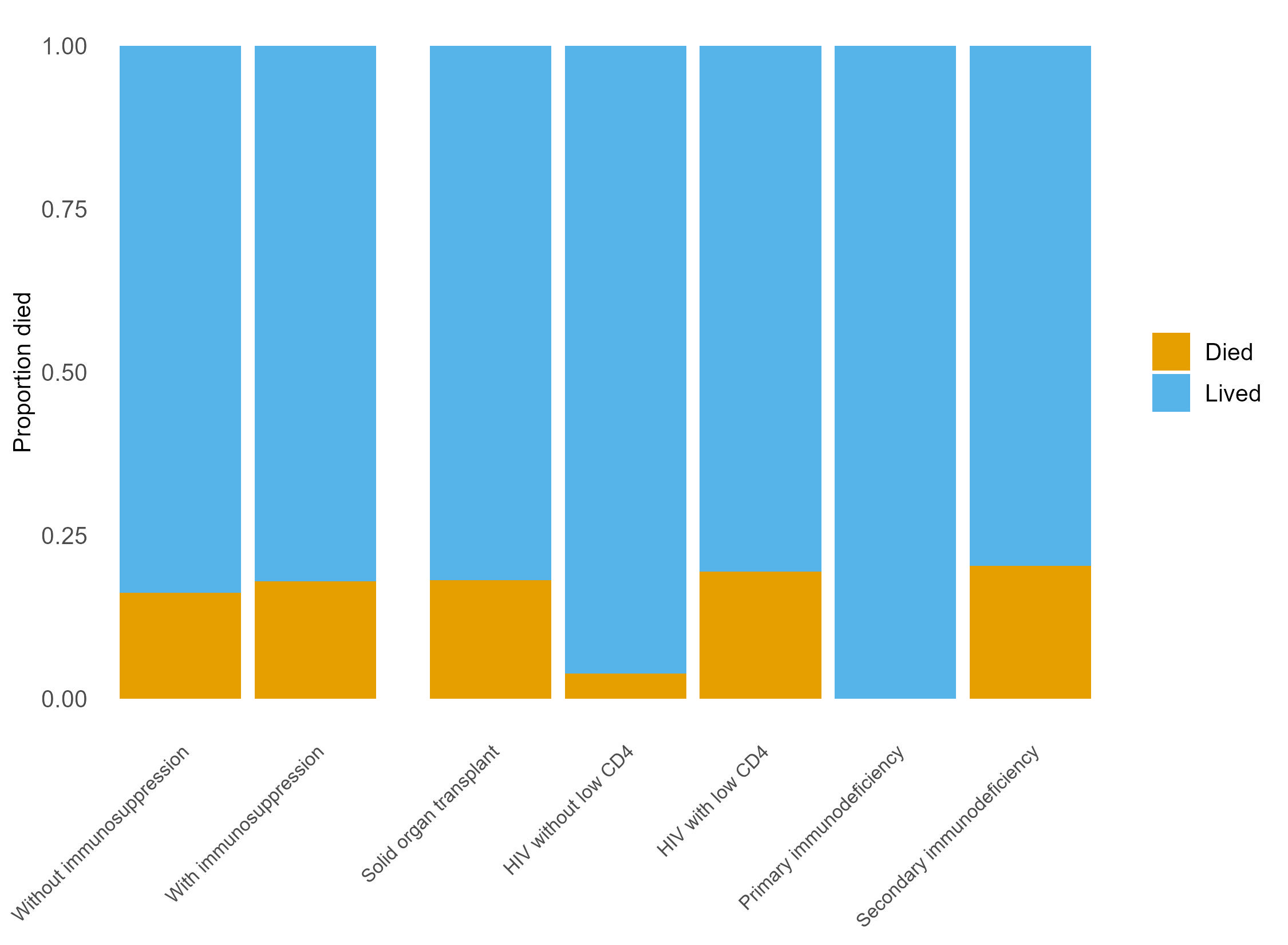

Supplement: S1 Fig — From 03/01/2020 to 05/31/2022, 10713 adult patients were hospitalized with COVID-19 at Northwestern Medicine. Of these patients, 9952 were hospitalized for at least two days and were categorized as without immunosuppression or with immunosuppression. Those with immunosuppression were further categorized as solid organ transplant, HIV, primary immunodeficiency, or secondary immunodeficiency based on the presence of diagnosis codes and manual adjudication. People with HIV were further categorized as without low CD4 or with low CD4 based on their absolute CD4 cell count closest in time to hospitalization. (TIF) [file pone.0330110.s001.tif]
