# Supplementary material for: Immunosuppression variably impacts outcomes for patients hospitalized with COVID-19: A retrospective cohort study
Source: PLoS One. 2025 Aug 8;20(8):e0330110. doi: 10.1371/journal.pone.0330110 (PMC12334029; doi:10.1371/journal.pone.0330110)
Supplement: S1 Table — (DOCX) [file pone.0330110.s002.docx]

**S1 Table. Full regression analysis results.**

Univariate model comparing non-exposure and exposure

|  |  | Regression coefficient | Standard error | 95% confidence interval | p-value |
| --- | --- | --- | --- | --- | --- |
| In-hospital mortality |  |  |  |  |  |
|  | With immunosuppression | 0.2886639 | 0.09409804 | 3.067693 | 2.16E-03 |
| Admitted to ICU |  |  |  |  |  |
|  | With immunosuppression | 0.5373473 | 0.07353169 | 7.307696 | 2.72E-13 |
| Low flow oxygenation |  |  |  |  |  |
|  | With immunosuppression | 0.1085806 | 0.08231685 | 1.319057 | 1.87E-01 |
| Noninvasive ventilation |  |  |  |  |  |
|  | With immunosuppression | 0.2250081 | 0.0741949 | 3.032663 | 2.42E-03 |
| Invasive ventilation |  |  |  |  |  |
|  | With immunosuppression | 0.5368594 | 0.10341869 | 5.191125 | 2.09E-07 |
| Mean hospitalization length |  |  |  |  |  |
|  | With immunosuppression | 0.3058044 | 0.029254685 | 10.453178 | 1.92E-25 |
| Mean length of ICU stay |  |  |  |  |  |
|  | With immunosuppression | 0.264535 | 0.097929832 | 2.701271 | 6.95E-03 |

Univariate model comparing non-exposure and exposure sub-groups

|  |  | Regression coefficient | Standard error | 95% confidence interval | p-value |
| --- | --- | --- | --- | --- | --- |
| In-hospital mortality |  |  |  |  |  |
|  | Solid organ transplant | 0.23057418 | 0.12698781 | 1.815719 | 6.94E-02 |
|  | HIV without low CD4 | -0.70149414 | 0.59986428 | -1.1694214 | 2.42E-01 |
|  | HIV with low CD4 | 0.53819425 | 0.37728452 | 1.4264944 | 1.54E-01 |
|  | Primary immunodeficiency | -0.93508412 | 1.02765003 | -0.9099247 | 3.63E-01 |
|  | Secondary immunodeficiency | 0.47188755 | 0.14665311 | 3.2177125 | 1.29E-03 |
| Admitted to ICU |  |  |  |  |  |
|  | Solid organ transplant | 0.62144164 | 0.09666851 | 6.4285843 | 1.29E-10 |
|  | HIV without low CD4 | -0.25743604 | 0.37805297 | -0.6809523 | 4.96E-01 |
|  | HIV with low CD4 | 0.52556747 | 0.3186293 | 1.6494637 | 9.91E-02 |
|  | Primary immunodeficiency | -0.16757971 | 0.57226363 | -0.2928366 | 7.70E-01 |
|  | Secondary immunodeficiency | 0.53565159 | 0.12109565 | 4.4233761 | 9.72E-06 |
| Low flow oxygenation |  |  |  |  |  |
|  | Solid organ transplant | 0.05660925 | 0.10831557 | 0.5226327 | 6.01E-01 |
|  | HIV without low CD4 | -0.36558752 | 0.33019943 | -1.1071719 | 2.68E-01 |
|  | HIV with low CD4 | -0.53685925 | 0.31863399 | -1.6848775 | 9.20E-02 |
|  | Primary immunodeficiency | 1.7501943 | 1.02747315 | 1.7033966 | 8.85E-02 |
|  | Secondary immunodeficiency | 0.32276923 | 0.1445572 | 2.2328132 | 2.56E-02 |
| Noninvasive ventilation |  |  |  |  |  |
|  | Solid organ transplant | 0.16890373 | 0.09938812 | 1.6994358 | 8.92E-02 |
|  | HIV without low CD4 | -0.95186277 | 0.44244242 | -2.1513822 | 3.14E-02 |
|  | HIV with low CD4 | -0.35142499 | 0.36299726 | -0.9681202 | 3.33E-01 |
|  | Primary immunodeficiency | 1.68719456 | 0.53277598 | 3.1667992 | 1.54E-03 |
|  | Secondary immunodeficiency | 0.42013056 | 0.11986776 | 3.5049506 | 4.57E-04 |
| Invasive ventilation |  |  |  |  |  |
|  | Solid organ transplant | 0.71904558 | 0.12866556 | 5.5884852 | 2.29E-08 |
|  | HIV without low CD4 | -1.36290352 | 1.00945076 | -1.3501436 | 1.77E-01 |
|  | HIV with low CD4 | 0.71651073 | 0.41567668 | 1.7237213 | 8.48E-02 |
|  | Primary immunodeficiency | 1.14729364 | 0.57295864 | 2.0024022 | 4.52E-02 |
|  | Secondary immunodeficiency | 0.26881286 | 0.18582166 | 1.4466174 | 1.48E-01 |
| Mean hospitalization length |  |  |  |  |  |
|  | Solid organ transplant | 0.30326181 | 0.038917691 | 7.7923896 | 7.23E-15 |
|  | HIV without low CD4 | 0.0225507 | 0.12916236 | 0.1745919 | 8.61E-01 |
|  | HIV with low CD4 | 0.06747912 | 0.127622446 | 0.5287403 | 5.97E-01 |
|  | Primary immunodeficiency | 0.72553878 | 0.200323199 | 3.621841 | 2.94E-04 |
|  | Secondary immunodeficiency | 0.35810521 | 0.048422602 | 7.3954144 | 1.52E-13 |
| Mean length of ICU stay |  |  |  |  |  |
|  | Solid organ transplant | 0.43209558 | 0.126087982 | 3.4269371 | 6.19E-04 |
|  | HIV without low CD4 | -1.20802588 | 0.560262941 | -2.1561767 | 3.12E-02 |
|  | HIV with low CD4 | -0.25775684 | 0.420801744 | -0.6125375 | 5.40E-01 |
|  | Primary immunodeficiency | 0.95495319 | 0.839529719 | 1.1374859 | 2.55E-01 |
|  | Secondary immunodeficiency | 0.15013476 | 0.160114987 | 0.9376684 | 3.48E-01 |

Multivariate model comparing non-exposure and exposure

|  |  | Regression coefficient | Standard error | 95% confidence interval | p-value |
| --- | --- | --- | --- | --- | --- |
| In-hospital mortality |  |  |  |  |  |
|  | With immunosuppression | 5.75E-01 | 9.99E-02 | 5.75E+00 | 8.97E-09 |
|  | Age at admission | 4.57E-02 | 2.04E-03 | 2.24E+01 | 7.12E-111 |
|  | Diabetes | 1.54E-01 | 6.24E-02 | 2.47E+00 | 1.34E-02 |
|  | Obesity | 6.88E-02 | 7.54E-02 | 9.12E-01 | 3.62E-01 |
|  | Omicron era | -8.15E-02 | 9.23E-02 | -8.82E-01 | 3.78E-01 |
|  | Pre-Delta era | -6.82E-02 | 7.73E-02 | -8.82E-01 | 3.78E-01 |
|  | Remdesivir | 2.33E-01 | 6.09E-02 | 3.82E+00 | 1.31E-04 |
|  | Paxlovid | 1.66E+00 | 1.45E+00 | 1.14E+00 | 2.53E-01 |
| Admitted to ICU |  |  |  |  |  |
|  | With immunosuppression | 4.93E-01 | 7.58E-02 | 6.50E+00 | 7.94E-11 |
|  | Age at admission | 8.46E-03 | 1.29E-03 | 6.57E+00 | 4.96E-11 |
|  | Diabetes | 3.82E-01 | 4.80E-02 | 7.97E+00 | 1.64E-15 |
|  | Obesity | 1.14E-01 | 5.48E-02 | 2.09E+00 | 3.69E-02 |
|  | Omicron era | 2.71E-02 | 6.90E-02 | 3.92E-01 | 6.95E-01 |
|  | Pre-Delta era | -2.91E-01 | 5.79E-02 | -5.02E+00 | 5.04E-07 |
|  | Remdesivir | 2.51E-01 | 4.70E-02 | 5.34E+00 | 9.31E-08 |
|  | Paxlovid | -1.08E+01 | 1.38E+02 | -7.81E-02 | 9.38E-01 |
| Low flow oxygenation |  |  |  |  |  |
|  | With immunosuppression | -1.19E-01 | 9.41E-02 | -1.26E+00 | 2.07E-01 |
|  | Age at admission | 2.88E-02 | 1.36E-03 | 2.12E+01 | 9.99E-100 |
|  | Diabetes | 3.23E-01 | 5.76E-02 | 5.60E+00 | 2.09E-08 |
|  | Obesity | 6.28E-01 | 6.74E-02 | 9.32E+00 | 1.21E-20 |
|  | Omicron era | -1.46E-01 | 8.14E-02 | -1.79E+00 | 7.29E-02 |
|  | Pre-Delta era | 2.34E-01 | 6.85E-02 | 3.41E+00 | 6.42E-04 |
|  | Remdesivir | 2.06E+00 | 6.85E-02 | 3.00E+01 | 7.65E-198 |
|  | Paxlovid | 1.10E+01 | 1.37E+02 | 8.08E-02 | 9.36E-01 |
| Noninvasive ventilation |  |  |  |  |  |
|  | With immunosuppression | 8.94E-02 | 8.10E-02 | 1.10E+00 | 2.70E-01 |
|  | Age at admission | 1.39E-02 | 1.36E-03 | 1.02E+01 | 2.49E-24 |
|  | Diabetes | 3.97E-01 | 4.88E-02 | 8.12E+00 | 4.49E-16 |
|  | Obesity | 6.42E-01 | 5.45E-02 | 1.18E+01 | 4.79E-32 |
|  | Omicron era | -4.17E-01 | 7.38E-02 | -5.65E+00 | 1.58E-08 |
|  | Pre-Delta era | -1.08E-01 | 5.87E-02 | -1.83E+00 | 6.67E-02 |
|  | Remdesivir | 1.32E+00 | 4.76E-02 | 2.77E+01 | 1.76E-169 |
|  | Paxlovid | -9.93E+00 | 1.38E+02 | -7.22E-02 | 9.42E-01 |
| Invasive ventilation |  |  |  |  |  |
|  | With immunosuppression | 5.19E-01 | 1.06E-01 | 4.91E+00 | 9.03E-07 |
|  | Age at admission | -1.18E-04 | 1.96E-03 | -6.01E-02 | 9.52E-01 |
|  | Diabetes | 2.93E-01 | 7.25E-02 | 4.04E+00 | 5.41E-05 |
|  | Obesity | 1.19E-01 | 8.08E-02 | 1.48E+00 | 1.39E-01 |
|  | Omicron era | -5.29E-01 | 1.18E-01 | -4.47E+00 | 7.76E-06 |
|  | Pre-Delta era | 2.70E-02 | 8.62E-02 | 3.14E-01 | 7.54E-01 |
|  | Remdesivir | 2.75E-01 | 7.15E-02 | 3.84194924 | 1.22E-04 |
|  | Paxlovid | -9.68E+00 | 2.29E+02 | -0.04221133 | 9.66E-01 |
| Mean hospitalization length |  |  |  |  |  |
|  | With immunosuppression | 0.28186112 | 0.028450456 | 9.9070863 | 4.96E-23 |
|  | Age at admission | 0.005803545 | 0.000434048 | 13.3707342 | 2.01E-40 |
|  | Diabetes | 0.1596701 | 0.017174566 | 9.2968929 | 1.75E-20 |
|  | Obesity | 0.035454599 | 0.019392453 | 1.8282679 | 6.75E-02 |
|  | Omicron era | -0.15746291 | 0.02496557 | -6.3072028 | 2.96E-10 |
|  | Pre-Delta era | 0.031008229 | 0.02053205 | 1.5102354 | 1.31E-01 |
|  | Remdesivir | 0.32447056 | 0.016619518 | 19.5234635 | 2.54E-83 |
|  | Paxlovid | 0.144939408 | 0.559229632 | 0.2591769 | 7.96E-01 |
| Mean length of ICU stay |  |  |  |  |  |
|  | With immunosuppression | 0.200231526 | 0.099305853 | 2.0163114 | 4.39E-02 |
|  | Age at admission | -0.008067012 | 0.001970004 | -4.0949224 | 4.35E-05 |
|  | Diabetes | 0.119857943 | 0.065876687 | 1.8194288 | 6.90E-02 |
|  | Obesity | 0.129711818 | 0.075349426 | 1.7214706 | 8.53E-02 |
|  | Omicron era | -0.387201156 | 0.094032702 | -4.1177287 | 3.94E-05 |
|  | Pre-Delta era | -0.116324494 | 0.080290654 | -1.4487925 | 1.48E-01 |
|  | Remdesivir | 0.148922912 | 0.064943377 | 2.2931193 | 2.19E-02 |
|  | Paxlovid | N/A | N/A | N/A | N/A |

Multivariate model comparing non-exposure and exposure sub-groups

|  |  | Regression coefficient | Standard error | 95% confidence interval | p-value |
| --- | --- | --- | --- | --- | --- |
| In-hospital mortality |  |  |  |  |  |
|  | Solid organ transplant | 5.64E-01 | 1.34E-01 | 4.212047138 | 2.53E-05 |
|  | HIV without low CD4 | -1.05E-01 | 6.10E-01 | -0.171992805 | 8.63E-01 |
|  | HIV with low CD4 | 1.17E+00 | 3.90E-01 | 2.991798827 | 2.77E-03 |
|  | Primary immunodeficiency | -7.97E-01 | 1.04E+00 | -0.766617734 | 4.43E-01 |
|  | Secondary immunodeficiency | 6.19E-01 | 1.54E-01 | 4.030258779 | 5.57E-05 |
|  | Age at admission | 4.58E-02 | 2.05E-03 | 22.32479156 | 2.12E-110 |
|  | Diabetes | 1.56E-01 | 6.27E-02 | 2.488302173 | 1.28E-02 |
|  | Obesity | 7.21E-02 | 7.55E-02 | 0.9549381 | 3.40E-01 |
|  | Omicron era | -7.97E-02 | 9.24E-02 | -0.862233498 | 3.89E-01 |
|  | Pre-Delta era | -6.77E-02 | 7.73E-02 | -0.875714395 | 3.81E-01 |
|  | Remdesivir | 2.34E-01 | 6.10E-02 | 3.832004025 | 1.27E-04 |
|  | Paxlovid | 1.66E+00 | 1.45E+00 | 1.141648356 | 2.54E-01 |
| Admitted to ICU |  |  |  |  |  |
|  | Solid organ transplant | 5.34E-01 | 9.98E-02 | 5.351677113 | 8.71E-08 |
|  | HIV without low CD4 | -1.90E-01 | 3.84E-01 | -0.495472296 | 6.20E-01 |
|  | HIV with low CD4 | 6.78E-01 | 3.23E-01 | 2.10057297 | 3.57E-02 |
|  | Primary immunodeficiency | -1.94E-01 | 5.76E-01 | -0.33625596 | 7.37E-01 |
|  | Secondary immunodeficiency | 5.17E-01 | 1.24E-01 | 4.183618636 | 2.87E-05 |
|  | Age at admission | 8.45E-03 | 1.29E-03 | 6.549013947 | 5.79E-11 |
|  | Diabetes | 3.82E-01 | 4.82E-02 | 7.915859004 | 2.46E-15 |
|  | Obesity | 1.16E-01 | 5.49E-02 | 2.109385782 | 3.49E-02 |
|  | Omicron era | 2.69E-02 | 6.90E-02 | 0.390206581 | 6.96E-01 |
|  | Pre-Delta era | -2.90E-01 | 5.79E-02 | -5.010695212 | 5.42E-07 |
|  | Remdesivir | 2.50E-01 | 4.70E-02 | 5.312884428 | 1.08E-07 |
|  | Paxlovid | -1.08E+01 | 1.38E+02 | -0.078084596 | 9.38E-01 |
| Low flow oxygenation |  |  |  |  |  |
|  | Solid organ transplant | -2.23E-01 | 1.25E-01 | -1.788280267 | 7.37E-02 |
|  | HIV without low CD4 | -4.71E-02 | 3.71E-01 | -0.127018896 | 8.99E-01 |
|  | HIV with low CD4 | -4.88E-01 | 3.65E-01 | -1.335474385 | 1.82E-01 |
|  | Primary immunodeficiency | 1.11E+00 | 1.07E+00 | 1.045032631 | 2.96E-01 |
|  | Secondary immunodeficiency | 6.27E-02 | 1.61E-01 | 0.388654374 | 6.98E-01 |
|  | Age at admission | 2.87E-02 | 1.36E-03 | 21.08371836 | 1.12E-98 |
|  | Diabetes | 3.29E-01 | 5.79E-02 | 5.688988612 | 1.28E-08 |
|  | Obesity | 6.23E-01 | 6.75E-02 | 9.224167977 | 2.86E-20 |
|  | Omicron era | -1.45E-01 | 8.14E-02 | -1.78076523 | 7.50E-02 |
|  | Pre-Delta era | 2.36E-01 | 6.85E-02 | 3.437713371 | 5.87E-04 |
|  | Remdesivir | 2.06E+00 | 6.86E-02 | 29.98717809 | 1.44E-197 |
|  | Paxlovid | 1.10E+01 | 1.37E+02 | 0.080794029 | 9.36E-01 |
| Noninvasive ventilation |  |  |  |  |  |
|  | Solid organ transplant | -1.99E-02 | 1.08E-01 | -0.184268563 | 8.54E-01 |
|  | HIV without low CD4 | -8.81E-01 | 4.70E-01 | -1.874791681 | 6.08E-02 |
|  | HIV with low CD4 | -2.09E-01 | 3.82E-01 | -0.547374129 | 5.84E-01 |
|  | Primary immunodeficiency | 1.41E+00 | 5.50E-01 | 2.553196645 | 1.07E-02 |
|  | Secondary immunodeficiency | 3.01E-01 | 1.30E-01 | 2.307051627 | 2.11E-02 |
|  | Age at admission | 1.36E-02 | 1.36E-03 | 9.992739768 | 1.64E-23 |
|  | Diabetes | 4.08E-01 | 4.91E-02 | 8.316681131 | 9.05E-17 |
|  | Obesity | 6.36E-01 | 5.46E-02 | 11.66535811 | 1.92E-31 |
|  | Omicron era | -4.13E-01 | 7.38E-02 | -5.601127442 | 2.13E-08 |
|  | Pre-Delta era | -1.07E-01 | 5.87E-02 | -1.820163396 | 6.87E-02 |
|  | Remdesivir | 1.32E+00 | 4.77E-02 | 27.70358178 | 6.32E-169 |
|  | Paxlovid | -9.93E+00 | 1.38E+02 | -0.072214813 | 9.42E-01 |
| Invasive ventilation |  |  |  |  |  |
|  | Solid organ transplant | 6.77E-01 | 1.32E-01 | 5.120636641 | 3.05E-07 |
|  | HIV without low CD4 | -1.35E+00 | 1.01E+00 | -1.333598103 | 1.82E-01 |
|  | HIV with low CD4 | 7.33E-01 | 4.18E-01 | 1.751382883 | 7.99E-02 |
|  | Primary immunodeficiency | 1.05E+00 | 5.76E-01 | 1.827653149 | 6.76E-02 |
|  | Secondary immunodeficiency | 2.92E-01 | 1.87E-01 | 1.558924978 | 1.19E-01 |
|  | Age at admission | -7.83E-06 | 1.97E-03 | -0.003974883 | 9.97E-01 |
|  | Diabetes | 2.84E-01 | 7.30E-02 | 3.890690148 | 1.00E-04 |
|  | Obesity | 1.27E-01 | 8.10E-02 | 1.572193317 | 1.16E-01 |
|  | Omicron era | -5.25E-01 | 1.18E-01 | -4.43877476 | 9.05E-06 |
|  | Pre-Delta era | 3.15E-02 | 8.62E-02 | 0.365178433 | 7.15E-01 |
|  | Remdesivir | 2.69E-01 | 7.16E-02 | 3.754351021 | 1.74E-04 |
|  | Paxlovid | -9.68E+00 | 2.29E+02 | -0.042211534 | 9.66E-01 |
| Mean hospitalization length |  |  |  |  |  |
|  | Solid organ transplant | 0.264520363 | 0.037865726 | 6.9857465 | 3.02E-12 |
|  | HIV without low CD4 | 0.101846016 | 0.123822322 | 0.8225174 | 4.11E-01 |
|  | HIV with low CD4 | 0.113583814 | 0.122349467 | 0.9283556 | 3.53E-01 |
|  | Primary immunodeficiency | 0.621859847 | 0.191938142 | 3.2398972 | 1.20E-03 |
|  | Secondary immunodeficiency | 0.337906436 | 0.046801202 | 7.2200376 | 5.58E-13 |
|  | Age at admission | 0.005747291 | 0.000434677 | 13.2219845 | 1.42E-39 |
|  | Diabetes | 0.161554268 | 0.017233978 | 9.3741718 | 8.50E-21 |
|  | Obesity | 0.033556206 | 0.019407909 | 1.7289965 | 8.38E-02 |
|  | Omicron era | -0.157226619 | 0.02496975 | -6.2966838 | 3.17E-10 |
|  | Pre-Delta era | 0.031285325 | 0.020529071 | 1.5239523 | 1.28E-01 |
|  | Remdesivir | 0.323583688 | 0.016623847 | 19.46503 | 7.63E-83 |
|  | Paxlovid | 0.144532382 | 0.559092601 | 0.2585124 | 7.96E-01 |
| Mean length of ICU stay |  |  |  |  |  |
|  | Solid organ transplant | 0.356769128 | 0.128161735 | 2.7837414 | 5.41E-03 |
|  | HIV without low CD4 | -1.266946846 | 0.556144901 | -2.2780877 | 2.28E-02 |
|  | HIV with low CD4 | -0.383894466 | 0.419086806 | -0.9160261 | 3.60E-01 |
|  | Primary immunodeficiency | 0.803577521 | 0.833920192 | 0.9636144 | 3.35E-01 |
|  | Secondary immunodeficiency | 0.123212701 | 0.160042093 | 0.7698768 | 4.41E-01 |
|  | Age at admission | -0.00797963 | 0.001971998 | -4.0464703 | 5.34E-05 |
|  | Diabetes | 0.109566179 | 0.066092883 | 1.6577606 | 9.75E-02 |
|  | Obesity | 0.133013807 | 0.075332896 | 1.7656803 | 7.76E-02 |
|  | Omicron era | -0.389846881 | 0.094095102 | -4.1431155 | 3.53E-05 |
|  | Pre-Delta era | -0.108664186 | 0.080293666 | -1.3533345 | 1.76E-01 |
|  | Remdesivir | 0.145507191 | 0.064874084 | 2.2429171 | 2.50E-02 |
|  | Paxlovid | N/A | N/A | N/A | N/A |
