# Supplementary material for: Immunosuppression variably impacts outcomes for patients hospitalized with COVID-19: A retrospective cohort study
Source: PLoS One. 2025 Aug 8;20(8):e0330110. doi: 10.1371/journal.pone.0330110 (PMC12334029; doi:10.1371/journal.pone.0330110)
Supplement: S2 Table — (DOCX) [file pone.0330110.s003.docx]

**S2 Table. Sex-stratified results, male.**

| **Male** | **Non-exposure** | **Exposure** | **Solid organ transplant** | **HIV+ normal CD4** | **HIV+ low CD4** | **Primary immunodeficiency** | **Secondary immunodeficiency** |
| --- | --- | --- | --- | --- | --- | --- | --- |
|  | **n = 4193** | **n = 512** | **n = 281** | **n = 26** | **n = 36** | **n = 7** | **n = 162** |
| **In-hospital mortality, n (%)** | 680 (16.22) | 92 (17.97) | 51 (18.15) | 1 (3.85) | 7 (19.44) | 0 (0.00) | 33 (20.37) |
| **Admitted to ICU, n (%)** | 1324 (31.58) | 212 (41.41) | 127 (45.20) | 5 (19.23) | 13 (36.11) | 1 (14.29) | 66 (40.74) |
| **Low flow oxygenation, n (%)** | 3381 (80.63) | 390 (76.17) | 217 (77.22) | 17 (65.38) | 23 (63.89) | 6 (85.71) | 127 (78.40) |
| **Non-invasive ventilation, n (%)** | 1655 (39.47) | 191 (37.30) | 101 (35.94) | 4 (15.38) | 8 (22.22) | 5 (71.43) | 73 (45.06) |
| **Invasive ventilation, n (%)** | 490 (11.69) | 78 (15.23) | 48 (17.08) | 1 (3.85) | 7 (19.44) | 1 (14.29) | 21 (12.96) |
| **Median hospitalization length (IQR)** | 6.00 (3.00, 11.0) | 7.00 (4.00, 15.0) | 7.00 (4.00, 15.0) | 5.50 (3.25, 11.8) | 5.50 (3.00, 10.5) | 13.0 (5.50, 21.5) | 7.50 (4.00, 15.0) |
| **Median ICU length of stay (IQR)** | 2.00 (1.00, 5.00) | 2.50 (1.00, 6.25) | 2.50 (1.00, 7.00) | 0.93 (0.24, 0.95) | 2.50 (0.93, 6.00) | 19.00 (19.00, 19.00) | 3.00 (1.75, 6.50) |
|  |  |  |  |  |  |  |  |
| **In-hospital mortality, unadjusted OR (95% CI)** | Ref | 1.13 (0.89, 1.43) | 1.15 (0.83, 1.56) | **0.21 (0.01, 0.98)** | 1.25 (0.50, 2.70) | 0.00 (NA, 509.89) | 1.32 (0.88, 1.93) |
| **Admitted to ICU, unadjusted OR (95% CI)** | Ref | **1.53 (1.27, 1.85)** | **1.79 (1.40, 2.28)** | 0.52 (0.17, 1.27) | 1.22 (0.60, 2.39) | 0.36 (0.02, 2.12) | **1.49 (1.08, 2.05)** |
| **Low flow oxygenation, unadjusted OR (95% CI)** | Ref | **0.77 (0.62, 0.96)** | 0.81 (0.61, 1.09) | 0.45 (0.21, 1.07) | **0.42 (0.22, 0.87)** | 1.44 (0.25, 27.23) | 0.87 (0.60, 1.29) |
| **Non-invasive ventilation, unadjusted OR (95% CI)** | Ref | 0.91 (0.75, 1.10) | 0.86 (0.67, 1.10) | **0.28 (0.08, 0.73)** | **0.44 (0.19, 0.92)** | 3.83 (0.83, 26.79) | 1.26 (0.92, 1.72) |
| **Invasive ventilation, unadjusted OR (95% CI)** | Ref | **1.36 (1.04, 1.75)** | **1.56 (1.11, 2.13)** | 0.30 (0.02, 1.43) | 1.82 (0.73, 3.96) | 1.26 (0.07, 7.39) | 1.13 (0.69, 1.76) |
| **Hospitalization length, unadjusted p-value** | Ref | **< 0.001** | **< 0.001** | 0.9422 | 0.9667 | **0.0415** | **0.0018** |
| **ICU length of stay, unadjusted p-value** | Ref | 0.1783 | **0.0484** | **0.0103** | 0.389 | 0.3536 | 0.4853 |
|  |  |  |  |  |  |  |  |
| **In-hospital mortality, adjusted OR (95% CI)** | Ref | **1.55 (1.20, 1.99)** | **1.65 (1.17, 2.28)** | 0.40 (0.02, 1.93) | 2.38 (0.93, 5.34) | 0.00 (NA, 337.60) | 1.52 (1.00, 2.25) |
| **Admitted to ICU, adjusted OR (95% CI)** | Ref | **1.43 (1.18, 1.73)** | **1.59 (1.24, 2.04)** | 0.50 (0.17, 1.24) | 1.27 (0.62, 2.50) | 0.35 (0.02, 2.09) | **1.46 (1.05, 2.01)** |
| **Low flow oxygenation, adjusted OR (95% CI)** | Ref | **0.65 (0.51, 0.83)** | **0.66 (0.48, 0.92)** | 0.60 (0.25, 1.49) | 0.50 (0.24, 1.08) | 0.66 (0.10, 13.09) | 0.69 (0.46, 1.05) |
| **Non-invasive ventilation, adjusted OR (95% CI)** | Ref | **0.81 (0.66, 0.99)** | **0.70 (0.53, 0.92)** | **0.31 (0.09, 0.87)** | 0.53 (0.22, 1.16) | 2.92 (0.58, 21.46) | 1.15 (0.82, 1.61) |
| **Invasive ventilation, adjusted OR (95% CI)** | Ref | 1.30 (0.99, 1.69) | **1.45 (1.03, 2.01)** | 0.30 (0.02, 1.41) | 1.72 (0.69, 3.74) | 1.20 (0.06, 7.11) | 1.14 (0.69, 1.78) |
| **Hospitalization length, adjusted p-value** | Ref | **< 0.001** | **0.0005** | 0.6085 | 0.7772 | 0.0618 | **0.0019** |
| **ICU length of stay, adjusted p-value** | Ref | 0.2716 | 0.0824 | **0.0069** | 0.3264 | 0.3953 | 0.5028 |
