# Supplementary material for: Immunosuppression variably impacts outcomes for patients hospitalized with COVID-19: A retrospective cohort study
Source: PLoS One. 2025 Aug 8;20(8):e0330110. doi: 10.1371/journal.pone.0330110 (PMC12334029; doi:10.1371/journal.pone.0330110)
Supplement: S3 Table — (DOCX) [file pone.0330110.s004.docx]

**S3 Table. Sex-stratified results, female.**

| **Female** | **Non-exposure** | **Exposure** | **Solid organ transplant** | **HIV+ normal CD4** | **HIV+ low CD4** | **Primary immunodeficiency** | **Secondary immunodeficiency** |
| --- | --- | --- | --- | --- | --- | --- | --- |
|  | **n = 4886** | **n = 361** | **n = 192** | **n = 15** | **n = 6** | **n = 10** | **n = 138** |
| **In-hospital mortality, n (%)** | 567 (11.60) | 61 (16.90) | 28 (14.58) | 2 (13.33) | 2 (33.33) | 1 (10.00) | 28 (20.29) |
| **Admitted to ICU, n (%)** | 1098 (22.47) | 123 (34.07) | 64 (33.33) | 4 (26.67) | 3 (50.00) | 3 (30.00) | 49 (35.51) |
| **Low flow oxygenation, n (%)** | 3296 (67.46) | 270 (74.79) | 136 (70.83) | 10 (66.67) | 3 (50.00) | 10 (100.00) | 111 (80.43) |
| **Non-invasive ventilation, n (%)** | 1137 (23.27) | 121 (33.52) | 62 (32.29) | 2 (13.33) | 2 (33.33) | 7 (70.00) | 48 (34.78) |
| **Invasive ventilation, n (%)** | 318 (6.51) | 47 (13.02) | 31 (16.15) | 0 (0.00) | 0 (0.00) | 3 (30.00) | 13 (9.42) |
| **Median hospitalization length (IQR)** | 5.00 (3.00, 8.00) | 7.00 (4.00, 15.0) | 6.50 (4.00, 13.0) | 4.00 (3.00, 8.00) | 4.00 (3.00, 6.50) | 12.0 (5.00, 27.8) | 7.00 (5.00, 16.0) |
| **Median ICU length of stay (IQR)** | 2.00 (1.00, 5.00) | 2.50 (1.00, 7.00) | 2.00 (1.00, 8.00) | 2.50 (1.75, 3.50) | 3.00 (2.00, 8.00) | 8.00 (5.50, 13.00) | 2.00 (0.93, 6.00) |
|  |  |  |  |  |  |  |  |
| **In-hospital mortality, unadjusted OR (95% CI)** | Ref | **1.55 (1.15, 2.05)** | 1.30 (0.85, 1.93) | 1.17 (0.18, 4.26) | 3.81 (0.53, 19.56) | 0.85 (0.05, 4.52) | **1.94 (1.25, 2.92)** |
| **Admitted to ICU, unadjusted OR (95% CI)** | Ref | **1.78 (1.42, 2.23)** | **1.72 (1.26, 2.34)** | 1.25 (0.35, 3.68) | 3.45 (0.64, 18.66) | 1.48 (0.32, 5.33) | **1.90 (1.32, 2.70)** |
| **Low flow oxygenation, unadjusted OR (95% CI)** | Ref | **1.43 (1.12, 1.84)** | 1.17 (0.86, 1.62) | 0.96 (0.34, 3.10) | 0.48 (0.09, 2.61) | Undefined | **1.98 (1.32, 3.09)** |
| **Non-invasive ventilation, unadjusted OR (95% CI)** | Ref | **1.66 (1.32, 2.08)** | **1.57 (1.15, 2.13)** | 0.51 (0.08, 1.84) | 1.65 (0.23, 8.46) | **7.69 (2.14, 35.74)** | **1.76 (1.22, 2.50)** |
| **Invasive ventilation, unadjusted OR (95% CI)** | Ref | **2.15 (1.53, 2.95)** | **2.77 (1.82, 4.07)** | Undefined | Undefined | **6.16 (1.32, 22.26)** | 1.49 (0.80, 2.57) |
| **Hospitalization length, unadjusted p-value** | Ref | **< 0.001** | **< 0.001** | 0.7639 | 0.7859 | **0.0015** | **< 0.001** |
| **ICU length of stay, unadjusted p-value** | Ref | **0.0175** | 0.0054 | 0.6992 | 0.964 | 0.3866 | 0.5682 |
|  |  |  |  |  |  |  |  |
| **In-hospital mortality, adjusted OR (95% CI)** | Ref | **2.11 (1.54, 2.85)** | **1.89 (1.20, 2.86)** | 2.11 (0.32, 8.07) | **9.97 (1.32, 54.58)** | 1.07 (0.06, 5.95) | **2.34 (1.47, 3.62)** |
| **Admitted to ICU, adjusted OR (95% CI)** | Ref | **1.73 (1.36, 2.19)** | **1.64 (1.18, 2.25)** | 1.38 (0.37, 4.21) | 4.58 (0.83, 25.22) | 1.40 (0.30, 5.15) | **1.85 (1.27, 2.67)** |
| **Low flow oxygenation, adjusted OR (95% CI)** | Ref | 1.09 (0.82, 1.46) | 0.82 (0.56, 1.20) | 1.31 (0.39, 4.72) | 0.20 (0.03, 1.42) | Undefined | **1.61 (1.01, 2.66)** |
| **Non-invasive ventilation, adjusted OR (95% CI)** | Ref | **1.44 (1.12, 1.86)** | 1.37 (0.97, 1.92) | 0.51 (0.08, 2.09) | 1.47 (0.19, 8.52) | **5.75 (1.52, 27.80)** | 1.49 (0.99, 2.20) |
| **Invasive ventilation, adjusted OR (95% CI)** | Ref | **2.21 (1.56, 3.07)** | **2.86 (1.86, 4.28)** | Undefined | Undefined | **5.42 (1.15, 19.84)** | 1.54 (0.82, 2.69) |
| **Hospitalization length, adjusted p-value** | Ref | **< 0.001** | **< 0.001** | 0.9989 | 0.8275 | **0.0054** | **< 0.001** |
| **ICU length of stay, adjusted p-value** | Ref | 0.0911 | **0.0276** | 0.6336 | 0.8841 | 0.4949 | 0.7959 |
