# Supplementary material for: Immunosuppression variably impacts outcomes for patients hospitalized with COVID-19: A retrospective cohort study
Source: PLoS One. 2025 Aug 8;20(8):e0330110. doi: 10.1371/journal.pone.0330110 (PMC12334029; doi:10.1371/journal.pone.0330110)
Supplement: S4 Table — (DOCX) [file pone.0330110.s005.docx]

**S4 Table. Hospitalization length and ICU length of stay stratified by discharge disposition.**

|  | **Non-exposure** | **Exposure** | **Solid organ transplant** | **HIV+ normal CD4** | **HIV+ low CD4** | **Primary immunodeficiency** | **Secondary immunodeficiency** |
| --- | --- | --- | --- | --- | --- | --- | --- |
|  | **n = 7832** | **n = 720** | **n = 394** | **n = 38** | **n = 33** | **n = 16** | **n = 239** |
| **Median hospitalization length to discharge alive (IQR)** | 5.00 (3.00, 8.00) | 6.00 (4.00, 12.00) | 6.00 (3.00, 11.75) | 5.00 (3.00, 8.00) | 5.00 (3.00, 10.00) | 11.50 (5.00, 27.75) | 7.00 (4.00, 14.00) |
| **Unadjusted p-value** | Ref | **< 0.001** | **< 0.001** | 0.716 | 0.555 | **< 0.001** | **< 0.001** |
| **Adjusted p-value** | Ref | **< 0.001** | **< 0.001** | 0.399 | 0.412 | **< 0.001** | **< 0.001** |
|  |  |  |  |  |  |  |  |
|  | n = 1409 | n = 174 | n = 94 | n = 7 | n = 9 | n = 4 | n = 60 |
| **Median ICU length of stay to discharge alive (IQR)** | 2.00 (1.00, 5.00) | 2.00 (1.00, 4.00) | 2.00 (1.00, 4.00) | 0.95 (0.59, 1.50) | 2.00 (1.00, 4.00) | 13.00 (6.75, 18.25) | 2.00 (1.00, 4.00) |
| **Unadjusted p-value** | Ref | 0.626 | 0.745 | **0.0381** | 0.762 | **0.015** | 0.215 |
| **Adjusted p-value** | Ref | 0.972 | 0.443 | **0.0273** | 0.965 | **0.0156** | 0.27 |
|  |  |  |  |  |  |  |  |
|  | n = 1247 | n = 153 | n = 79 | n = 3 | n = 9 | n = 1 | n = 61 |
| **Median hospitalization length to in-hospital mortality (IQR)** | 8.00 (5.00, 15.00) | 15.00 (7.00, 29.00) | 18.00 (8.50, 30.00) | 11.00 (7.00, 19.50) | 6.00 (4.00, 18.00) | 14.00 (14.00, 14.00) | 13.00 (6.00, 20.00) |
| **Unadjusted p-value** | Ref | **< 0.001** | **< 0.001** | 0.797 | 0.573 | 0.561 | **< 0.001** |
| **Adjusted p-value** | Ref | **< 0.001** | **< 0.001** | 0.945 | 0.214 | 0.599 | **0.0336** |
|  |  |  |  |  |  |  |  |
|  | n = 517 | n = 84 | n = 45 | n = 2 | n = 6 | n = 0 | n = 31 |
| **Median ICU length of stay to in-hospital mortality (IQR)** | 3.00 (1.00, 8.00) | 6.00 (2.00, 12.00) | 8.00 (3.00, 16.00) | 4.00 (3.50, 4.50) | 4.50 (1.31, 10.50) | Undefined | 5.00 (1.00, 10.00) |
| **Unadjusted p-value** | Ref | **< 0.001** | **< 0.001** | 0.643 | 0.889 | Undefined | 0.323 |
| **Adjusted p-value** | Ref | **0.0377** | **0.00335** | 0.817 | 0.714 | Undefined | 0.843 |
