# Supplementary material for: Immunosuppression variably impacts outcomes for patients hospitalized with COVID-19: A retrospective cohort study
Source: PLoS One. 2025 Aug 8;20(8):e0330110. doi: 10.1371/journal.pone.0330110 (PMC12334029; doi:10.1371/journal.pone.0330110)
Supplement: Attachment — Submitted filename: PONE-D-24-51256-Academic editor-comments.pdf [file pone.0330110.s007.pdf]

PLOS ONE

Outcomes for patients hospitalized with COVID-19 vary by pre-existing immunosuppressive condition: A retrospective cohort study

--Manuscript Draft--

**Manuscript Number:**	PONE-D-24-51256
**Article Type:**	Research Article
**Full Title:**	Outcomes for patients hospitalized with COVID-19 vary by pre-existing immunosuppressive condition: A retrospective cohort study
**Short Title:**	Outcomes for patients hospitalized with COVID-19 vary by pre-existing immunosuppressive condition
**Corresponding Author:**	Theresa Walunas<br>Northwestern University Feinberg School of Medicine<br>Chicago, IL UNITED STATES OF AMERICA
**Keywords:**	COVID-19; Immunosuppression; hospitalization; solid organ transplant; HIV/AIDS; primary immunodeficiency; secondary immunodeficiency
**Abstract:**	Background: Adults with solid organ transplant, HIV, primary immunodeficiency, and secondary immunodeficiency are more likely to develop severe COVID-19 than immunocompetent adults. Less is known about differences in outcomes for adults with these conditions hospitalized with COVID-19.<br>Methods: A retrospective cohort study of adults hospitalized with COVID-19 at Northwestern Medicine between 03/01/2020 and 05/31/2022 was performed. Regression analyses were performed comparing hospitalization length, oxygenation requirements, ICU admission/length of stay, and in-hospital mortality among patients with no immunosuppression (n = 9079), solid organ transplant (n = 473), HIV without (n = 41) and with low CD4 cell count (n = 42), primary immunodeficiency (n = 17), and secondary immunodeficiency (n = 300).<br>Results: Immunocompetent patients were hospitalized for 8.6 days on average. Patients with solid organ transplant, primary immunodeficiency, and secondary immunodeficiency had longer mean hospitalization lengths of 13.5, 18.2, and 12.9 days (p < 0.001 for each comparison). Necessity for invasive ventilation was higher in the solid organ transplant (16.3%) and primary immunodeficiency groups (23.5%) by day 28 compared to immunocompetent patients (8.8%, p < 0.001; p = 0.046). Patients with HIV did not have longer hospitalization or worse oxygenation than immunocompetent patients, but those with low CD4 cell count had higher 28-day cumulative incidence of ICU admission (38.1%) and death (9.5%) than immunocompetent patients (26.7% ICU admission, p = 0.028; 5.5% death, p = 0.014).<br>Conclusions: Patients with solid organ transplant, primary immunodeficiency, and secondary immunodeficiency had worse outcomes than immunocompetent patients following hospitalization with COVID-19. Patients with HIV had similar outcomes as immunocompetent patients unless CD4 cell count was low.
**Order of Authors:**	Vijeeth Guggilla
	Jennifer A Pacheco
	Alexandre M Carvalho
	Grant R Whitmer
	Anna Pawlowski
	Jodi L Johnson
	Catherine A Gao
	Chad J Achenbach
	Theresa L Walunas
**Opposed Reviewers:**	
**Additional Information:**	

Powered by Editorial Manager® and ProduXion Manager® from Aries Systems Corporation

Question	Response
**Financial Disclosure**<br><br>Enter a financial disclosure statement that describes the sources of funding for the work included in this submission. Review the submission guidelines for detailed requirements. View published research articles from *PLOS ONE* for specific examples.<br><br>This statement is required for submission and **will appear in the published article** if the submission is accepted. Please make sure it is accurate.<br><br>**Funded studies**<br>Enter a statement with the following details:<br>• Initials of the authors who received each award<br>• Grant numbers awarded to each author<br>• The full name of each funder<br>• URL of each funder website<br>• Did the sponsors or funders play any role in the study design, data collection and analysis, decision to publish, or preparation of the manuscript?<br><br>Did you receive funding for this work?	Yes
Please add funding details.<br>as follow-up to "**Financial Disclosure**<br><br>Enter a financial disclosure statement that describes the sources of funding for the work included in this submission. Review the submission guidelines for detailed requirements. View published research articles from *PLOS ONE* for specific examples.<br><br>This statement is required for submission and **will appear in the published article** if the submission is accepted. Please make sure it is accurate.	TLW received grant number CO-US-540-6535 from Gilead Sciences (https://www.gilead.com/) for this work.<br><br>The funders reviewed the final manuscript.

Powered by Editorial Manager® and ProduXion Manager® from Aries Systems Corporation

**Funded studies**<br>Enter a statement with the following details:<br>• Initials of the authors who received each award<br>• Grant numbers awarded to each author<br>• The full name of each funder<br>• URL of each funder website<br>• Did the sponsors or funders play any role in the study design, data collection and analysis, decision to publish, or preparation of the manuscript?<br><br>Did you receive funding for this work?"	
Please select the country of your main research funder (please select carefully as in some cases this is used in fee calculation).<br>as follow-up to "**Financial Disclosure**<br><br>Enter a financial disclosure statement that describes the sources of funding for the work included in this submission. Review the submission guidelines for detailed requirements. View published research articles from *PLOS ONE* for specific examples.<br><br>This statement is required for submission and **will appear in the published article** if the submission is accepted. Please make sure it is accurate.<br><br>**Funded studies**<br>Enter a statement with the following details:<br>• Initials of the authors who received each award<br>• Grant numbers awarded to each author<br>• The full name of each funder<br>• URL of each funder website<br>• Did the sponsors or funders play any role in the study design, data collection and analysis, decision to publish, or preparation of the manuscript?<br><br>Did you receive funding for this work?"	UNITED STATES - US
**Competing Interests**	The authors have declared that no competing interests exist.

Powered by Editorial Manager® and ProduXion Manager® from Aries Systems Corporation

Use the instructions below to enter a competing interest statement for this submission. On behalf of all authors, disclose any competing interests that could be perceived to bias this work—acknowledging all financial support and any other relevant financial or non-financial competing interests.<br><br>This statement is required for submission and **will appear in the published article** if the submission is accepted. Please make sure it is accurate and that any funding sources listed in your Funding Information later in the submission form are also declared in your Financial Disclosure statement.<br><br>View published research articles from *PLOS ONE* for specific examples.<br><br>**NO authors have competing interests**<br><br>Enter: *The authors have declared that no competing interests exist.*<br><br>**Authors with competing interests**<br><br>Enter competing interest details beginning with this statement:<br><br>*I have read the journal's policy and the authors of this manuscript have the following competing interests: [insert competing interests here]*<br><br>* typeset	
**Ethics Statement**<br><br>Enter an ethics statement for this submission. This statement is required if the study involved:<br><br>• Human participants<br>• Human specimens or tissue<br>• Vertebrate animals or cephalopods<br>• Vertebrate embryos or tissues	This was a retrospective study performed under a waiver of consent. This study was approved by the Northwestern University Institutional Review Board (STU00217318).

• Field research<br><br>Write "N/A" if the submission does not require an ethics statement.<br><br>General guidance is provided below. Consult the submission guidelines for detailed instructions. **Make sure that all information entered here is included in the Methods section of the manuscript.**	

Powered by Editorial Manager® and ProduXion Manager® from Aries Systems Corporation

**Format for specific study types**<br><br>**Human Subject Research (involving human participants and/or tissue)**<br>• Give the name of the institutional review board or ethics committee that approved the study<br>• Include the approval number and/or a statement indicating approval of this research<br>• Indicate the form of consent obtained (written/oral) or the reason that consent was not obtained (e.g. the data were analyzed anonymously)<br><br>**Animal Research (involving vertebrate animals, embryos or tissues)**<br>• Provide the name of the Institutional Animal Care and Use Committee (IACUC) or other relevant ethics board that reviewed the study protocol, and indicate whether they approved this research or granted a formal waiver of ethical approval<br>• Include an approval number if one was obtained<br>• If the study involved *non-human primates*, add *additional details* about animal welfare and steps taken to ameliorate suffering<br>• If anesthesia, euthanasia, or any kind of animal sacrifice is part of the study, include briefly which substances and/or methods were applied<br><br>**Field Research**<br><br>Include the following details if this study involves the collection of plant, animal, or other materials from a natural setting:<br>• Field permit number<br>• Name of the institution or relevant body that granted permission	
**Data Availability**<br><br>Authors are required to make all data underlying the findings described fully available, without restriction, and from the time of publication. PLOS allows rare exceptions to address legal and ethical concerns. See the PLOS Data Policy and FAQ for detailed information.	No - some restrictions will apply

A Data Availability Statement describing where the data can be found is required at submission. Your answers to this question constitute the Data Availability Statement and **will be published in the article**, if accepted.<br><br>**Important:** Stating 'data available on request from the author' is not sufficient. If your data are only available upon request, select 'No' for the first question and explain your exceptional situation in the text box.<br><br>Do the authors confirm that all data underlying the findings described in their manuscript are fully available without restriction?	
**Describe where the data may be found in full sentences. If you are copying our sample text, replace any instances of XXX with the appropriate details.**<br><br>• If the data are **held or will be held in a public repository**, include URLs, accession numbers or DOIs. If this information will only be available after acceptance, indicate this by ticking the box below. For example: *All XXX files are available from the XXX database (accession number(s) XXX, XXX.*).<br>• If the data are all contained **within the manuscript and/or Supporting Information files**, enter the following: *All relevant data are within the manuscript and its Supporting Information files.*<br>• If neither of these applies but you are able to provide **details of access elsewhere**, with or without limitations, please do so. For example:<br><br>*Data cannot be shared publicly because of [XXX]. Data are available from the XXX Institutional Data Access / Ethics Committee (contact via XXX) for researchers who meet the criteria for access to confidential data.*<br><br>*The data underlying the results presented in the study are available from (include the name of the third party*	Data cannot be shared publicly because data contain protected health information (including potentially identifying or sensitive patient information). Data are available from the corresponding author on reasonable request for researchers who meet the criteria for access to confidential data. Note that row-level data are access-controlled and cannot be provided publicly due to data use restrictions.

Powered by Editorial Manager® and ProduXion Manager® from Aries Systems Corporation

and contact information or URL).<br>• This text is appropriate if the data are owned by a third party and authors do not have permission to share the data.<br><br>* typeset	
Additional data availability information:	

Powered by Editorial Manager® and ProduXion Manager® from Aries Systems Corporation

Northwestern University
Feinberg School of Medicine
750 North Lake Shore Drive
10th Floor
Chicago, IL 60611-2927

Division of General Internal Medicine and Geriatrics
Department of Medicine

NORTHWESTERN UNIVERSITY

November 8, 2024

Emily Chenette, PhD
Editor-in-Chief, *PLOS ONE*

Dear Dr. Chenette:

Please find enclosed our manuscript, "Outcomes for patients hospitalized with COVID-19 vary by pre-existing immunosuppressive condition: A retrospective cohort study". We are submitting this paper for consideration as a Research Article in *PLOS ONE*.

Individuals who are immunosuppressed are more likely to develop severe COVID-19. However, there remains much to learn about outcomes of COVID-19 hospitalization in individuals with immunosuppressive conditions such as solid organ transplant, HIV, and primary and secondary immunodeficiency. For example, the evidence surrounding COVID-19 hospitalization outcomes among patients with solid organ transplant has been inconsistent, and there have been no studies comparing the outcomes of COVID-19 hospitalization among patients with primary and secondary immunodeficiency. Furthermore, while many prior studies of COVID-19 and immunosuppression have focused on mortality, there are fewer studies comparing other important COVID-19 hospital outcomes such as hospitalization length, ventilatory support, and ICU care. Finally, prior research on COVID-19 and immunosuppression has largely occurred through 2020 or 2021, with few studies in 2022.

This study aimed to address these gaps by leveraging real-world clinical data from electronic health records to compare hospitalization length, ventilatory support, ICU care, and mortality among patients with solid organ transplant, HIV, and primary and secondary immunodeficiency hospitalized with COVID-19 and immunocompetent patients hospitalized with COVID-19. The study showed that patients with solid organ transplant and primary and secondary immunodeficiency generally have worse outcomes than immunocompetent patients. People with HIV did not have worse outcomes on the surface, but stratifying by CD4 cell count found higher mortality in people with HIV with low CD4 cell count. To our knowledge, this is the first study of all four of these immunosuppressive conditions in the context of COVID-19 from a unified dataset. This study also had a large study population of 9952 total patients, of which 873 had an immunosuppressive condition.

Our work adds to previous publications in your esteemed journal such as Suárez-García et al's study "In-hospital mortality among immunosuppressed patients with COVID-19: Analysis from a national cohort in Spain" and Swan et al's study "Hospitalization and survival of solid organ transplant recipients with coronavirus disease 2019: A propensity matched cohort study", providing additional insight into the complex relationship between immunosuppression and outcomes of COVID-19. We therefore believe *PLOS ONE* to be a great fit for this manuscript.

This manuscript is not being considered for publication at any other journal, nor has any of the data in this manuscript been previously published elsewhere. All authors have contributed significantly to the work, reviewed the final manuscript, and approved its submission to *PLOS ONE*.

The corresponding author for this manuscript:

Theresa L. Walunas, PhD, MS, FAMIA
Center for Health Information Partnerships
Northwestern University Feinberg School of Medicine
625 N. Michigan Avenue, 15th Floor
Chicago, IL 60611, USA

Telephone: (312) 503-3691
Email: t-walunas@northwestern.edu

We sincerely appreciate your review of our manuscript and look forward to feedback from the *PLOS ONE* editorial team and reviewers.

Kind Regards,

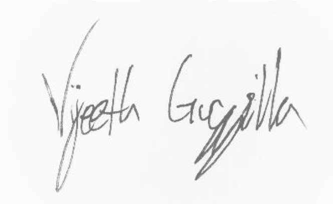

Vijeeth Guggilla, BA
Doctoral Research Assistant, MD-PhD Candidate

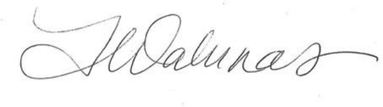

Theresa L. Walunas, PhD, MS, FAMIA
Associate Professor of Medicine
Associate Director, Center for Health Information Partnerships

Manuscript

Click here to access/download;Manuscript;VG_Manuscript_PLOS.docx

Outcomes for patients hospitalized with COVID-19 vary by pre-existing immunosuppressive condition: A retrospective cohort study

Vijeeth Guggilla[1,2], Jennifer A Pacheco[3], Alexandre M Carvalho[2], Grant R Whitmer[4], Anna E Pawlowski[5], Jodi L Johnson[6,7,8,9], Catherine A Gao[10], Chad J Achenbach[11,12], Theresa L Walunas[1,4,9,11*]

[1]Center for Health Information Partnerships, Northwestern University Feinberg School of Medicine, Chicago, Illinois, USA

[2]Northwestern University Feinberg School of Medicine, Chicago, Illinois, USA

[3]Center for Genetic Medicine, Northwestern University Feinberg School of Medicine, Chicago, Illinois, USA

[4]Department of Medicine, Northwestern University Feinberg School of Medicine, Chicago, Illinois, USA

[5]Northwestern Medicine Enterprise Data Warehouse, Northwestern University Feinberg School of Medicine, Chicago, Illinois, USA

[6]Robert H. Lurie Comprehensive Cancer Center of Northwestern University, Chicago, Illinois, USA

[7]Department of Pathology, Northwestern University Feinberg School of Medicine, Chicago, Illinois, USA

[8]Department of Dermatology, Northwestern University Feinberg School of Medicine, Chicago, Illinois, USA

[9]Department of Medical Social Sciences, Northwestern University Feinberg School of Medicine, Chicago, Illinois, USA

[10]Division of Pulmonary and Critical Care Medicine, Department of Medicine, Northwestern University Feinberg School of Medicine, Chicago, Illinois, USA

[11]Department of Preventive Medicine, Northwestern University Feinberg School of Medicine, Chicago, Illinois, USA

[12]Division of Infectious Diseases, Department of Medicine, Northwestern University Feinberg School of Medicine, Chicago, Illinois, USA

***** Corresponding author

E-mail: t-walunas@northwestern.edu (TLW)

Abstract

Background: Adults with solid organ transplant, HIV, primary immunodeficiency, and secondary immunodeficiency are more likely to develop severe COVID-19 than immunocompetent adults. Less is known about differences in outcomes for adults with these conditions hospitalized with COVID-19.

Methods: A retrospective cohort study of adults hospitalized with COVID-19 at Northwestern Medicine between 03/01/2020 and 05/31/2022 was performed. Regression analyses were performed comparing hospitalization length, oxygenation requirements, ICU admission/length of stay, and in-hospital mortality among patients with no immunosuppression (n = 9079), solid organ transplant (n = 473), HIV without (n = 41) and with low CD4 cell count (n = 42), primary immunodeficiency (n = 17), and secondary immunodeficiency (n = 300).

Results: Immunocompetent patients were hospitalized for 8.6 days on average. Patients with solid organ transplant, primary immunodeficiency, and secondary immunodeficiency had longer mean hospitalization lengths of 13.5, 18.2, and 12.9 days ($p < 0.001$ for each comparison). Necessity for invasive ventilation was higher in the solid organ transplant (16.3%) and primary immunodeficiency groups (23.5%) by day 28 compared to immunocompetent patients (8.8%, $p < 0.001$; $p = 0.046$). Patients with HIV did not have longer hospitalization or worse oxygenation than immunocompetent patients, but those with low CD4 cell count had higher 28-day cumulative incidence of ICU admission (38.1%) and death (9.5%) than immunocompetent patients (26.7% ICU admission, $p = 0.028$; 5.5% death, $p = 0.014$).

Conclusions: Patients with solid organ transplant, primary immunodeficiency, and secondary immunodeficiency had worse outcomes than immunocompetent patients following hospitalization with COVID-19. Patients with HIV had similar outcomes as immunocompetent patients unless CD4 cell count was low.

Introduction

Severity of coronavirus disease 2019 (COVID-19), the respiratory illness resulting from infection with SARS-CoV-2, ranges from asymptomatic to extensive lung damage requiring hospitalization. Since 2020, the COVID-19 pandemic has had a significant impact on healthcare and society around the world. As of April 6, 2024, COVID-19 has caused over 6.9 million hospitalizations and nearly 1.9 million deaths in the United States alone [1,2]. While severe COVID-19 cases have substantially decreased thanks to vaccination and rising population immunity, periodic surges in infections continue, and risks continue to be elevated for populations with certain underlying health conditions [3].

One such at-risk population includes individuals with pre-existing immunosuppressive conditions. A systematic review comparing patients with solid organ transplant (SOT) to the general population hospitalized with COVID-19 showed higher rates of intensive care and in-hospital mortality in the SOT group [4]. HIV has also been identified as a risk factor for poor outcomes in the context of COVID-19. Multiple systematic reviews and cohort studies have found significantly higher COVID-19 associated mortality in people with HIV (PWH) compared to those without HIV [5–9]. One of these cohort

studies, performed in US National COVID Cohort Collaborative (N3C) data, also showed higher odds of COVID-19 hospitalization among PWH [10]. Finally, individuals with primary (PI) and secondary immunodeficiencies (SI) in the United Kingdom Primary Immunodeficiency Network who developed COVID-19 as well as a cohort of individuals with PI in New York City with COVID-19 had higher inpatient mortality than the general population [11,12]. A systematic review performed by the Centers for Disease Control and Prevention (CDC) found similar results as well as higher rates of ICU admission for patients with PI who developed COVID-19 [13].

While this evidence suggests that SOT, HIV, PI, and SI increase the risks associated with COVID-19, there is also evidence to the contrary. For example, three different cohort studies in the US, Spain, and Italy have found that compared to patients from the general population, patients with SOT hospitalized with COVID-19 had no differences in mortality, with the US-based study suggesting that differences in age and comorbidities rather than SOT status itself are contributors to any observed differences in mortality [14–16]. Kalil and Florescu further discuss the inconsistent results that have been observed in patients with SOT hospitalized with COVID-19 [17]. With regard to HIV, studies have shown increased mortality with COVID-19 co-infection, but there have also been a number of cohort studies in PWH hospitalized with COVID-19 that have shown no differences in mortality compared to those without HIV [18–31]. This suggests important differences in outcomes when comparing populations hospitalized with COVID-19.

Clearly, the immune milieu impacts COVID-19 progression and severity, but much remains to be understood about the impact of specific immunosuppressive conditions

on outcomes of severe COVID-19 among hospitalized patients. For example, there have been no studies comparing the outcomes of COVID-19 hospitalization in individuals with PI and SI, and the evidence surrounding COVID-19 hospitalization outcomes among patients with SOT has been inconsistent. While COVID-19 hospitalization outcomes have been extensively studied in PWH, the influence of low CD4 cell count on outcomes of COVID-19 has been less clear [19,23,29,30,32,33]. In addition, there have been no studies comparing all four of these immunosuppressive conditions in a single cohort study with a unified approach. Furthermore, although many studies have focused on mortality, there are fewer studies comparing other COVID-19 hospital outcomes such as hospitalization length, ventilatory support, and ICU care. Finally, prior research on COVID-19 and immunosuppression occurred through 2020 or 2021, with few studies in 2022, the era of Omicron SARS-CoV-2 subvariants and high levels of vaccine-induced and natural immunity.

We aimed to address these gaps to improve our knowledge of COVID-19 epidemiology and outcomes for different immunosuppressed populations, which is crucial for public health guidance [34]. Many of these gaps exist because studying COVID-19 hospitalization outcomes for patients with specific immunosuppressive conditions has been challenging due to the rarity of individuals with such conditions at a given healthcare institution. Given these challenges, leveraging data available in the electronic health record (EHR) is a robust strategy for studying how severe COVID-19 outcomes differ among individuals with different immunosuppressive conditions. In this study, we compared differences in hospitalization length, oxygenation requirements, ICU admission/length of stay, and in-hospital mortality for adults with a history of SOT,

HIV, PI, or SI hospitalized with COVID-19 compared to adults with no history of immunosuppression hospitalized with COVID-19.

Methods

Ethics statement

This was a retrospective study performed under a waiver of consent. This study was approved by the Northwestern University Institutional Review Board (STU00217318).

Study design and setting

We performed a single-health system retrospective cohort study utilizing data from the Northwestern Medicine (NM) Enterprise Data Warehouse, which serves as an integrated repository for all the EHR data of patients who have received care within the NM health system.

Study population

We included adults (at least 18 years of age) hospitalized with COVID-19 at NM hospitals between 03/01/2020 and 05/31/2022. Patients were only included if they ever received a positive COVID-19 laboratory test (positive polymerase chain reaction on nasopharyngeal or lower respiratory tract specimens, positive SARS-CoV-2 antigenic test) and were also hospitalized in the period encompassing one day before to seven days after the laboratory test result. Patients were excluded if they were not hospitalized

or if they tested positive for COVID-19 more than one day after hospitalization. Patients were also excluded if they were hospitalized for less than two days to minimize inclusion of individuals hospitalized for less severe disease and individuals hospitalized for reasons other than COVID-19 who were quickly discharged.

~~Definitions and d~~data collection

International Classification of Diseases, 9th and 10th Revisions (ICD-9 and ICD-10) diagnosis codes that patients had received prior to their hospitalization with COVID-19 were extracted from the EHR and used to initially assign each patient to either no immunosuppression or one of four immunosuppression groups: SOT (includes kidney transplant, liver transplant, heart transplant, or lung transplant), HIV, PI (includes antibody deficiency, combined immunodeficiency, common variable immunodeficiency, ataxia-telangiectasia, phagocyte deficiency, or complement deficiency), or SI (includes patients taking immunosuppressant medication for autoimmune disease and patients who have received hematopoietic stem cell transplants). For each patient assigned to HIV, their absolute CD4 cell count closest to the time of hospitalization including the period encompassing seven days after hospitalization was also retrieved from the EHR. PWH with absolute CD4 cell count less than or equal to 350 cells/µL were categorized as having low CD4 cell count. We performed chart review on all patients assigned to an immunosuppression group to accurately classify categorization of their immunosuppression. Patients found to have a different immunosuppression category than expected based on ICD coding were reassigned to the appropriate group. Patients found to fall into multiple categories of immunosuppression were assigned to only one group per the following hierarchy: SOT, HIV, PI, and SI. Confirmatory ~~chart review~~ was

guided by clinical experts and performed either by physicians or medical students with physician support.

Age at admission, sex assigned at birth, race, ethnicity, insurance status, and most recent body mass index (BMI) measurement were extracted from the EHR data for each included patient. ICD-9 and ICD-10 diagnosis codes were used to define whether patients had a history of diabetes. Dates of hospital and ICU admission and discharge, in-hospital oxygenation information, and in-hospital all-cause mortality were also extracted from the EHR. Dates of hospital admission were used to assign SARS-CoV-2 variant era with categorization into three groups based on calendar time of when a specific variant of concern (VOC) accounted for greater than 50% of circulating viruses determined per sequencing surveillance. The eras falling within our study timeline were defined as: Pre-Delta (ancestral and early variants of concern including alpha and gamma, 03/01/2020 to 06/30/2021), Delta (07/01/2021 to 12/31/2021), and Omicron (01/01/2022 to 05/31/2022).

All data were first accessed on 05/21/2023 for research purposes. Authors had access to information that could identify individual participants during and after data collection.

~~Outcomes and statistical analysis~~

Dates of admission and discharge were used to determine hospitalization length. Rate of ICU admission and length of ICU stay were determined based on ICU transfer in or admission date and ICU transfer out or discharge date. Multivariate linear regression analyses adjusting for age at admission, diabetes, obesity, and SARS-CoV-2 variant era as covariates were performed to assess the effect of immune group status on mean

hospitalization length and mean length of ICU stay. For hospitalization length, the logarithm transform of the data was used to achieve normality of the residuals. We chose to adjust for these covariates because age, diabetes, and obesity have all been identified as the strongest risk factors for death in patients hospitalized with COVID-19, and because of changes in pandemic landscape over time with the rise and fall of different VOCs [35,36].

Multivariate logistic regression analyses were performed adjusting for the same covariates to assess the effect of immune group status on administration of low flow oxygen, non-invasive ventilation (including high-flow nasal cannula), and invasive ventilation (intubation, ECMO) within 14 days of hospitalization and within 28 days of hospitalization. Fourteen and 28-day cumulative incidence of ICU admission and all-cause mortality censored at discharge were also assessed. For all logistic regression analyses, influential values were assessed and determined not to impact results. There was no multicollinearity observed across all the analyses. The threshold for significance was set at 5% with 95% confidence intervals. Full regression analysis results are in S1 Table.

All analyses were performed in the R software environment, Version 4.3.1 (2023-06-16).

Results

Characteristics of the study ~~population~~

We identified 9952 patients hospitalized for at least two days with COVID-19 at NM between 03/01/2020 and 05/31/2022. Of these, 1123 patients were chart reviewed and confirmed to have been hospitalized with COVID-19. After further categorization based on ICD codes and CD4 cell count (for PWH) and chart review, we analyzed 9079 patients with no immunosuppression, 473 patients with SOT, 41 PWH without low CD4 cell count, 42 PWH with low CD4 cell count, 17 patients with PI, and 300 patients with SI (Fig 1). Complete data were available for the demographic variables and outcomes we assessed.

Fig 1. Flowchart of study population identification and grouping. From 03/01/2020 to 05/31/2022, 10713 patients were hospitalized with COVID-19 at Northwestern Medicine. Of these patients, 9952 were hospitalized for at least two days and were categorized as no immunosuppression, solid organ transplant, HIV, primary immunodeficiency, or secondary immunodeficiency based on the presence of diagnosis codes and chart review. Of the 83 patients with HIV, 41 were further categorized as without low CD4 and 42 were categorized as with low CD4 based on their absolute CD4 cell count closest in time to hospitalization.

The mean age of the included cohort was 61.1 years. The mean ages of the immune group categories were similar except that PWH without and with low CD4 cell count had lower mean ages of 50.2 years and 51.7 years, respectively. Among the included cohort, 47.3% were assigned male and 52.7% were assigned female at birth. Similar

proportions of men to women were seen in the immune group categories except that 63.4% and >75% of PWH with and without low CD4 cell count were men, respectively. Detailed descriptive statistics of the COVID-19 study population and its respective immune status categories are shown in Table 1.

Table 1. Descriptive statistics of the COVID-19 study population across immune status groups.

	Total	No immunosuppression	Solid organ transplant	HIV without low CD4	HIV with low CD4	Primary immunodeficiency	Secondary immunodeficiency
	**n = 9952**	**n = 9079**	**n = 473**	**n = 41**	**n = 42**	**n = 17**	**n = 300**
**Mean age at admission, y (SD)**	61.1 (19.0)	61.5 (19.3)	55.7 (14.2)	50.2 (14.8)	51.7 (12.7)	59.8 (13.1)	60.6 (14.7)
**Sex assigned at birth, n (%)**							
Men	4705 (47.3)	4193 (46.2)	281 (59.4)	26 (63.4)	--	--	162 (54.0)
Women	5247 (52.7)	4886 (53.8)	192 (40.6)	15 (36.6)	--	--	138 (46.0)
**Race, n (%)**							
American Indian or Alaska Native	39 (0.4)	34 (0.4)	--	0 (0.0)	0 (0.0)	0 (0.0)	--
Asian	336 (3.4)	302 (3.3)	23 (4.9)	--	--	0 (0.0)	9 (3.0)
Black or African American	1682 (16.9)	1462 (16.1)	112 (23.7)	26 (63.4)	19 (45.2)	--	62 (20.7)
Native Hawaiian or Other Pacific Islander	31 (0.3)	29 (0.3)	--	0 (0.0)	0 (0.0)	0 (0.0)	--
White	6710 (67.4)	6214 (68.4)	257 (54.3)	13 (31.7)	17 (40.5)	13 (76.5)	196 (65.3)
Unknown	1154 (11.6)	1038 (11.4)	76 (16.1)	--	--	--	31 (10.3)
**Ethnicity, n (%)**							
Hispanic or Latino	1960 (19.7)	1770 (19.5)	--	--	--	--	--
Not Hispanic or Latino	7758 (78.0)	7097 (78.2)	328 (69.3)	36 (87.8)	34 (81.0)	14 (82.4)	249 (83.0)
Unknown	234 (2.4)	212 (2.3)	--	--	--	--	--
**Insurance status, n (%)**							
Commercial	2828 (28.4)	2569 (28.3)	123 (26.0)	--	--	--	105 (35.0)
Medicare	1587 (16.0)	1480 (16.3)	51 (10.8)	14 (34.2)	13 (31.0)	0 (0.0)	--
Medicaid	5028 (50.5)	4540 (50.0)	286 (60.5)	14 (34.2)	14 (33.3)	--	165 (55.0)
Self-Pay	509 (5.1)	490 (5.4)	13 (2.8)	--	--	--	--
**SARS-CoV-2 variant era, n (%)**							
Pre-Delta	5826 (58.5)	5363 (59.1)	241 (51.0)	26 (63.4)	26 (61.9)	10 (58.8)	160 (53.3)
Delta	2039 (20.5)	1885 (20.8)	86 (18.2)	--	--	--	49 (16.3)
Omicron	2035 (20.5)	1785 (19.7)	144 (30.4)	--	--	--	87 (29.0)

**Mean BMI (SD)**	30.8 (8.3)	30.9 (8.4)	28.4 (5.8)	30.1 (9.3)	26.3 (8.2)	28.2 (6.5)	29.9 (7.7)
**Diabetes, n (%)**	3553 (35.7)	3161 (34.8)	264 (55.8)	18 (43.9)	10 (23.8)	--	96 (32.0)

Counts less than 10 and greater than zero are not reported (denoted by "--") to protect patient privacy.

Hospitalization outcomes

Patients with SOT were hospitalized significantly longer (13.5 days) than patients with no immunosuppression (8.6 days, $p < 0.001$) when adjusting for age at admission, diabetes, obesity, and SARS-CoV-2 variant era. The use of invasive ventilation at day 14 (14.8%) and day 28 (16.3%) was also significantly greater compared to patients with no immunosuppression (7.8% at day 14; 8.8% at day 28; $p < 0.001$ for both comparisons). Use of low flow oxygenation, use of noninvasive ventilation, and mortality were not significantly different compared to patients with no immunosuppression (Table 2).

Table 2. Hospitalization length, oxygenation requirements, and mortality of the COVID-19 study population across immune status groups.

	**No immunosuppression**	**Solid organ transplant**	Adjusted p-value	**HIV without low CD4**	Adjusted p-value	**HIV with low CD4**	Adjusted p-value	**Primary immunodeficiency**	Adjusted p-value	**Secondary immunodeficiency**	Adjusted p-value
**Mean hospitalization length, days (SD)**	8.6 (11.6)	13.5 (21.7)	**< 0.001**	8.9 (9.3)	0.416	9.9 (12.2)	0.211	18.2 (16.9)	**< 0.001**	12.9 (16.6)	**< 0.001**
**Low flow oxygenation, day 14, n (%)**	6084 (67.0)	300 (63.4)	0.975	20 (48.8)	0.157	21 (50.0)	0.256	15 (88.2)	0.077	205 (68.3)	0.403
**Low flow oxygenation, day 28, n (%)**	6169 (68.0)	307 (64.9)	0.865	22 (53.7)	0.366	21 (50.0)	0.207	16 (94.1)	**0.043**	214 (71.3)	0.140
**Noninvasive ventilation, day 14, n (%)**	2430 (26.8)	129 (27.3)	0.324	4 (9.8)	**0.047**	8 (19.1)	0.699	11 (64.7)	**0.001**	97 (32.3)	**0.014**
**Noninvasive ventilation, day 28, n (%)**	2513 (27.7)	142 (30.0)	0.074	5 (12.2)	0.082	10 (23.8)	0.804	12 (70.6)	**< 0.001**	104 (34.7)	**0.003**
**Invasive ventilation, day 14, n (%)**	710 (7.8)	70 (14.8)	**< 0.001**	1 (2.4)	0.226	5 (11.9)	0.282	2 (11.8)	0.566	26 (8.7)	0.428
**Invasive ventilation, day 28, n (%)**	800 (8.8)	77 (16.3)	**< 0.001**	1 (2.4)	0.188	7 (16.7)	0.062	4 (23.5)	**0.046**	31 (10.3)	0.222
**All-cause mortality, day 14, n (%)**	285 (3.1)	7 (1.5)	0.583	0 (0.0)	0.973	2 (4.8)	0.082	1 (5.9)	0.345	11 (3.7)	0.196
**All-cause mortality, day 28, n (%)**	502 (5.5)	25 (5.3)	0.063	0 (0.0)	0.957	4 (9.5)	**0.014**	1 (5.9)	0.739	22 (7.3)	**0.025**

Adjusted p-values are from multivariate regression analyses with age at admission, diabetes, obesity, and SARS-CoV-2 variant era as covariates with no immunosuppression as the reference, and p-values < 0.05 are significant (bold).

Significantly fewer PWH without low CD4 cell count required non-invasive ventilation at day 14 after hospitalization (9.8%) compared to those with no immunosuppression (26.8%, $p = 0.047$). There were no significant differences in any other hospitalization outcomes for this group compared to the no immunosuppression group (Table 2). PWH with low CD4 cell count had significantly higher 28-day cumulative incidence of death (9.5%) compared to patients with no immunosuppression (5.5%, $p = 0.014$). There were no significant differences in any other hospitalization outcomes for this group compared to the no immunosuppression group (Table 2).

Patients with PI were hospitalized significantly longer (18.2 days) than patients with no immunosuppression (8.6 days, $p < 0.001$). The use of low flow oxygenation by day 28 (94.1%), noninvasive ventilation by day 14 (64.7%) and day 28 (70.6%), and invasive ventilation by day 28 (23.5%) were also significantly higher compared to patients with no immunosuppression (68.0% low flow oxygenation by day 28, $p = 0.043$; 26.8% noninvasive ventilation by day 14, $p = 0.001$; 27.7% noninvasive ventilation by day 28, $p < 0.001$; 8.8% invasive ventilation by day 28, $p = 0.046$). There were no significant differences in low flow oxygenation and invasive ventilation by day 14 or in mortality compared to the no immunosuppression group (Table 2).

Patients with SI were hospitalized significantly longer (12.9 days) than patients with no immunosuppression (8.6 days, $p < 0.001$). The use of noninvasive ventilation by day 14 (32.3%) and day 28 (34.7%) was also significantly greater compared to patients with no immunosuppression (26.8% by day 14, $p = 0.014$; 27.7% by day 28, $p = 0.003$). These patients also had significantly higher mortality by day 28 after hospitalization (7.3%) compared to patients with no immunosuppression (5.5%, $p = 0.025$). There were no

significant differences in any other hospitalization outcomes for this group compared to the no immunosuppression group (Table 2).

ICU-related outcomes

Compared to patients with no immunosuppression admitted to the ICU (26.7%), patients with SOT (40.4%, $p < 0.001$), PWH with low CD4 cell count (38.1%, $p = 0.028$), and patients with SI (38.3%, $p < 0.001$) all had a significantly higher proportion of individuals admitted to the ICU when adjusting for age at admission, diabetes, obesity, and SARS-CoV-2 variant era. The proportions of PWH without low CD4 cell count and patients with PI admitted to the ICU were not significantly different compared to patients with no immunosuppression. Patients with SOT also had a significantly longer length of ICU stay (5.9 days) compared to patients with no immunosuppression (4.4 days, $p = 0.003$). In contrast, PWH without low CD4 cell count had a significantly shorter length of ICU stay (1.7 days, $p = 0.023$). PWH with low CD4 cell count, patients with PI, and patients with SI did not have a significantly different mean length of ICU stay compared to patients with no immunosuppression (Table 3).

Table 3. ICU admission and length of ICU stay of the COVID-19 study population across immune status groups.

	**No immunosuppression**	**Solid organ transplant**	Adjusted p-value	**HIV without low CD4**	Adjusted p-value	**HIV with low CD4**	Adjusted p-value	**Primary immunodeficiency**	Adjusted p-value	**Secondary immunodeficiency**	Adjusted p-value
**Admitted to ICU, n (%)**	2422 (26.7)	191 (40.4)	**< 0.001**	9 (22.0)	0.626	16 (38.1)	**0.028**	4 (23.5)	0.841	115 (38.3)	**< 0.001**
**Mean length of ICU stay, days (SD)**	4.4 (5.7)	5.9 (7.2)	**0.003**	1.7 (1.5)	**0.023**	4.8 (5.4)	0.370	12.0 (7.8)	0.340	5.7 (7.2)	0.383

Adjusted p-values are from multivariate regression analyses with age at admission, diabetes, obesity, and SARS-CoV-2 variant era as covariates with no immunosuppression as the reference, and p-values < 0.05 are significant (bold).

Discussion

This study compared the outcomes of COVID-19 hospitalization for patients with different immunosuppressive conditions (SOT, HIV, PI, and SI). We identified 10713 patients hospitalized with COVID-19 at NM between 03/01/2020 and 05/31/2022, and we categorized 873 of these patients as having a pre-existing immunosuppressive condition. The proportions of individuals found to have each immunosuppressive condition of interest followed our expectations based on the estimated prevalence of these conditions in the US and the fact that we were likely to oversample for such conditions given NM is an academic health system [37,38]. The differences observed in mean age and sex distribution for PWH compared to the total study population and other immune groups were expected based on the known demographics and age distribution for PWH [39].

Patients with SOT had the poorest experiences following hospitalization with COVID-19. Not only was their hospitalization length significantly longer than patients with no immunosuppression, but they also had higher rates of invasive ventilation, higher rates of ICU admission, and longer length of ICU stay. This aligns with a systematic review of patients with SOT compared to patients from the general population hospitalized with COVID-19 which also showed higher rates of intensive care in patients with SOT [4]. However, while this systematic review found higher mortality in patients with SOT, we observed no differences in mortality. This could be due to our study adjusting for comorbidities such as age, diabetes, and obesity. Our results also align with findings from three separate cohort studies that compared patients with SOT and patients from

the general population hospitalized with COVID-19 and found no differences in mortality. Notably, Moreno-Torres et al. and Rinaldi et al. adjusted for comorbidities similar to those in our analysis [14–16].

Hospitalization was also longer in patients with PI and SI. This aligns with findings from a systematic review performed by the CDC showing greater morbidity for patients with PI who develop COVID-19 [13]. Unlike previous results from a cohort study in the United Kingdom Primary Immunodeficiency Network (UKPIN), we observed no differences in mortality for patients with PI compared to patients with no immunosuppression, although we did observe increased mortality in patients with SI [11]. However, it is important to note that the UKPIN study examined patients who developed COVID-19 and not necessarily patients hospitalized with COVID-19. In the SI group, a significantly higher proportion of individuals were admitted to the ICU compared to patients with no immunosuppression, but mean length of ICU stay was not significantly different. Overall, our results add knowledge of the relationship between PI and SI and COVID-19 by specifically examining patients hospitalized with COVID-19, which no other studies have done in the context of PI or SI.

Unlike the other immunosuppression groups, PWH without low CD4 cell count had similar or better outcomes than patients with no immunosuppression. This was expected as these patients were likely well engaged in care including consistent use of combination antiretroviral therapy that led to great immune recovery. PWH with low CD4 cell count had a higher rate of ICU admission and higher mortality by day 28 after hospitalization. A cohort study in Italy of PWH with low CD4 cell count hospitalized with COVID-19 also found higher mortality in this group compared to the general population.

However, while this study found that PWH with low CD4 cell count were hospitalized for significantly longer, we did not observe any significant differences in hospitalization length, oxygenation requirements, or length of ICU stay compared to patients with no immunosuppression [30]. It was interesting that we observed a similar trend in mortality despite hospitalization outcomes that were otherwise not worse compared to immunocompetent patients. This could be due to low CD4 cell count being a marker for less engagement in care or due to these patients dying from something other than COVID-19.

Patients with SOT may have particularly poor outcomes due to long-term higher levels of immunosuppression from medications to minimize organ rejection. Corticosteroids, which suppress general inflammation, and calcineurin inhibitors, which suppress T cell development, are most commonly employed. In contrast, PWH experience a loss of adaptive immunity, and PI and SI can lead to a loss of adaptive or innate immunity based on the specific immunodeficiency a patient has. Clearly, different mechanisms of immunosuppression can result in varying severity and outcomes of COVID-19 hospitalization. However, it is important to keep in mind that both our groups of PWH and patients with PI were small, and we may not be adequately powered to detect true differences.

Our study is limited by potential data missingness and misclassification inherent to EHR data. Firstly, because the EHR is a real-world data source, it is not guaranteed to capture all the desired information about a patient. For example, information can be fragmented across multiple systems and may not always be entered accurately. Thus, the presence or absence of information in the EHR may not always reflect reality

regarding factors like immunosuppression status. In addition, while we chart reviewed all patients in the immunosuppression groups, we did not exhaustively chart review all variables for the entire study population. Thus, because we only pulled patients who were coded as immunosuppressed in the EHR, we recognize that there may be patients in the no immunosuppression group who should be in one of the immunosuppression groups. This is especially true for our SI group, which represents a broad category of immunosuppression. Another limitation was our focus on hospitalization EHR data and not assessing outcomes beyond discharge such as hospital readmission or death outside of the hospital setting. Finally, our study is limited to a single health system and therefore may not be representative of the general population. This is especially important given NM is an academic health system with more complex, sicker, and better insured patients compared to community-based hospital systems.

Our next steps are to expand our study to include multi-health system EHR data. By applying the COVID-19 and immunosuppression definitions we developed at a larger population level, we can greatly increase our sample sizes for each immunosuppression group and gain a more representative sampling across the US. Because our present study only ascertained hospital outcomes, we also plan to study longer term outcomes such as one-year all-cause mortality or development of long COVID. This may provide further insight into the lasting effects of COVID-19, which can impact health well beyond hospital discharge [40].

In conclusion, our findings suggest that patients with immunosuppression from various causes have different outcomes after hospitalization with COVID-19. Our data have implications for healthcare providers and public health professionals responding to

COVID-19 and future pandemics of emerging infectious diseases. While public awareness of the COVID-19 pandemic may be waning, health systems should continue to monitor patients with SOT, HIV, PI, and SI given the possibility of worse outcomes compared to the general public following hospitalization with COVID-19.

References

1. CDC. COVID Data Tracker [Internet]. Centers for Disease Control and Prevention. 2020 [cited 2024 Apr 16]. Available from: https://covid.cdc.gov/covid-data-tracker

2. Pei S, Yamana TK, Kandula S, Galanti M, Shaman J. Burden and characteristics of COVID-19 in the United States during 2020. Nature. 2021 Oct;598(7880):338–41.

3. The Changing Threat of COVID-19 | CDC [Internet]. 2024 [cited 2024 Apr 16]. Available from: https://www.cdc.gov/ncird/whats-new/changing-threat-covid-19.html

4. Belsky JA, Tullius BP, Lamb MG, Sayegh R, Stanek JR, Auletta JJ. COVID-19 in immunocompromised patients: A systematic review of cancer, hematopoietic cell and solid organ transplant patients. J Infect. 2021 Mar 1;82(3):329–38.

5. Ssentongo P, Heilbrunn ES, Ssentongo AE, Advani S, Chinchilli VM, Nunez JJ, et al. Epidemiology and outcomes of COVID-19 in HIV-infected individuals: a systematic review and meta-analysis. Sci Rep. 2021 Mar 18;11(1):6283.

6. Danwang C, Noubiap JJ, Robert A, Yombi JC. Outcomes of patients with HIV and COVID-19 co-infection: a systematic review and meta-analysis. AIDS Res Ther. 2022 Jan 14;19(1):3.

7. Bhaskaran K, Rentsch CT, MacKenna B, Schultze A, Mehrkar A, Bates CJ, et al. HIV infection and COVID-19 death: a population-based cohort analysis of UK primary care data and linked national death registrations within the OpenSAFELY platform. Lancet HIV. 2021 Jan;8(1):e24–32.

8. Miyashita H, Kuno T. Prognosis of coronavirus disease 2019 (COVID-19) in patients with HIV infection in New York City. HIV Med. 2021 Jan;22(1):e1–2.

9. Western Cape Department of Health in collaboration with the National Institute for Communicable Diseases, South Africa. Risk Factors for Coronavirus Disease 2019 (COVID-19) Death in a Population Cohort Study from the Western Cape Province, South Africa. Clin Infect Dis Off Publ Infect Dis Soc Am. 2021 Oct 5;73(7):e2005–15.

10. Yang X, Sun J, Patel RC, Zhang J, Guo S, Zheng Q, et al. Associations between HIV infection and clinical spectrum of COVID-19: a population level analysis based on US National COVID Cohort Collaborative (N3C) data. Lancet HIV. 2021 Nov;8(11):e690–700.

11. Shields AM, Anantharachagan A, Arumugakani G, Baker K, Bahal S, Baxendale H, et al. Outcomes following SARS-CoV-2 infection in patients with primary and secondary immunodeficiency in the UK. Clin Exp Immunol. 2022 Sep 1;209(3):247–58.

12. Ho H en, Mathew S, Peluso MJ, Cunningham-Rundles C. Clinical outcomes and features of COVID-19 in patients with primary immunodeficiencies in New York City. J Allergy Clin Immunol Pract. 2021 Jan 1;9(1):490-493.e2.

13. Morford M, Green RF, Drzymalla E, Okasako-Schmucker DL, So CN, Hill AL, et al. Brief Summary of Findings on the Association Between Underlying Primary Immunodeficiency and Severe COVID-19 Outcomes. CDC COVID-19 Sci Brief.

14. Kates OS, Haydel BM, Florman SS, Rana MM, Chaudhry ZS, Ramesh MS, et al. Coronavirus Disease 2019 in Solid Organ Transplant: A Multicenter Cohort Study. Clin Infect Dis Off Publ Infect Dis Soc Am. 2021 Dec 6;73(11):e4090–9.

15. Rinaldi M, Bartoletti M, Bussini L, Pancaldi L, Pascale R, Comai G, et al. COVID-19 in solid organ transplant recipients: No difference in survival compared to general population. Transpl Infect Dis. 2021 Feb;23(1):e13421.

16. Moreno-Torres V, Martínez-Urbistondo M, Calderón-Parra J, Mills P, Muñoz-Serrano A, Arias-Milla A, et al. COVID-19 in hospitalized solid organ transplant recipients in a nationwide registry study. Int J Infect Dis IJID Off Publ Int Soc Infect Dis. 2023 Sep;134:154–9.

17. Kalil AC, Florescu DF. Mortality in solid organ transplant recipients hospitalized for COVID-19. Am J Transplant. 2022 Jan;22(1):12–3.

18. Karmen-Tuohy S, Carlucci PM, Zervou FN, Zacharioudakis IM, Rebick G, Klein E, et al. Outcomes Among HIV-Positive Patients Hospitalized With COVID-19. J Acquir Immune Defic Syndr 1999. 2020 Jun 12;10.1097/QAI.0000000000002423.

19. Flannery S, Schwartz R, Rasul R, Hirschwerk DA, Wallach F, Hirsch B, et al. A comparison of COVID-19 inpatients by HIV status. Int J STD AIDS. 2021 Oct;32(12):1149–56.

20. Durstenfeld MS, Sun K, Ma Y, Rodriguez F, Secemsky EA, Parikh RV, et al. Association of HIV infection with outcomes among adults hospitalized with COVID-19. AIDS Lond Engl. 2022 Mar 1;36(3):391–8.

21. Gagliardini R, Vergori A, Lorenzini P, Cicalini S, Pinnetti C, Mazzotta V, et al. Characteristics and Outcomes of COVID-19-Related Hospitalization among PLWH. J Clin Med. 2022 Mar 11;11(6):1546.

22. Ceballos ME, Ross P, Lasso M, Dominguez I, Puente M, Valenzuela P, et al. Clinical characteristics and outcomes of people living with HIV hospitalized with COVID-19: a nationwide experience. Int J STD AIDS. 2021 Apr;32(5):435–43.

23. Patel VV, Felsen UR, Fisher M, Fazzari MJ, Ginsberg MS, Beil R, et al. Clinical Outcomes and Inflammatory Markers by HIV Serostatus and Viral Suppression in a Large Cohort of Patients Hospitalized With COVID-19. J Acquir Immune Defic Syndr 1999. 2021 Feb 1;86(2):224–30.

24. Lee MJ, Snell LB, Douthwaite ST, Fidler S, Fitzgerald N, Goodwin L, et al. Clinical outcomes of patients with and without HIV hospitalized with COVID-19 in England during the early stages of the pandemic: a matched retrospective multi-centre analysis (RECEDE-C19 study). HIV Med. 2022 Feb;23(2):121–33.

25. Nagarakanti SR, Okoh AK, Grinberg S, Bishburg E. Clinical outcomes of patients with COVID-19 and HIV coinfection. J Med Virol. 2021 Mar;93(3):1687–93.

26. Faiz Z, Quazi MA, Vahil N, Barrows CM, Ikram HA, Nasrullah A, et al. COVID-19 and HIV: Clinical Outcomes among Hospitalized Patients in the United States. Biomedicines. 2023 Jul 5;11(7):1904.

27. Stoeckle K, Johnston CD, Jannat-Khah DP, Williams SC, Ellman TM, Vogler MA, et al. COVID-19 in Hospitalized Adults With HIV. Open Forum Infect Dis. 2020 Aug;7(8):ofaa327.

28. Díez C, Del Romero-Raposo J, Mican R, López JC, Blanco JR, Calzado S, et al. COVID-19 in hospitalized HIV-positive and HIV-negative patients: A matched study. HIV Med. 2021 Oct;22(9):867–76.

29. Chanda D, Minchella PA, Kampamba D, Itoh M, Hines JZ, Fwoloshi S, et al. COVID-19 Severity and COVID-19-Associated Deaths Among Hospitalized Patients with HIV Infection - Zambia, March-December 2020. MMWR Morb Mortal Wkly Rep. 2021 Jun 4;70(22):807–10.

30. Giacomelli A, Gagliardini R, Tavelli A, De Benedittis S, Mazzotta V, Rizzardini G, et al. Risk of COVID-19 in-hospital mortality in people living with HIV compared to general population according to age and CD4 strata: data from the ICONA network. Int J Infect Dis IJID Off Publ Int Soc Infect Dis. 2023 Nov;136:127–35.

31. Looha MA, Taraghikhah N, Amini M, Pormehr PS, Talaei N, Khodadoost M, et al. The impact of HIV on the risk of COVID-19 death among hospitalized patients. Hum Antibodies. 2022;30(4):165–75.

32. Nomah DK, Reyes-Urueña J, Díaz Y, Moreno S, Aceiton J, Bruguera A, et al. Sociodemographic, clinical, and immunological factors associated with SARS-CoV-2 diagnosis and severe COVID-19 outcomes in people living with HIV: a retrospective cohort study. Lancet HIV. 2021 Nov;8(11):e701–10.

33. Dandachi D, Geiger G, Montgomery MW, Karmen-Tuohy S, Golzy M, Antar AAR, et al. Characteristics, Comorbidities, and Outcomes in a Multicenter Registry of Patients With Human Immunodeficiency Virus and Coronavirus Disease 2019. Clin Infect Dis Off Publ Infect Dis Soc Am. 2021 Oct 5;73(7):e1964–72.

34. CDC Newsroom [Internet]. CDC. 2016 [cited 2024 Apr 17]. Available from: https://www.cdc.gov/media/releases/2024/p0301-respiratory-virus.html

35. Kompaniyets L. Underlying Medical Conditions and Severe Illness Among 540,667 Adults Hospitalized With COVID-19, March 2020–March 2021. Prev Chronic Dis [Internet]. 2021 [cited 2024 May 15];18. Available from: https://www.cdc.gov/pcd/issues/2021/21_0123.htm

36. Pennington AF, Kompaniyets L, Summers AD, Danielson ML, Goodman AB, Chevinsky JR, et al. Risk of Clinical Severity by Age and Race/Ethnicity Among Adults Hospitalized for COVID-19—United States, March–September 2020. Open Forum Infect Dis. 2021 Feb 1;8(2):ofaa638.

37. Boyle JM, Buckley RH. Population Prevalence of Diagnosed Primary Immunodeficiency Diseases in the United States. J Clin Immunol. 2007 Sep 1;27(5):497–502.

38. Estimated HIV incidence and prevalence in the United States, 2014–2018 [Internet]. [cited 2024 Apr 18]. Available from: https://stacks.cdc.gov/view/cdc/87841

39. HIV & AIDS Trends and U.S. Statistics Overview [Internet]. HIV.gov. [cited 2024 May 2]. Available from: https://www.hiv.gov/hiv-basics/overview/data-and-trends/statistics

40. Huang L, Li X, Gu X, Zhang H, Ren L, Guo L, et al. Health outcomes in people 2 years after surviving hospitalisation with COVID-19: a longitudinal cohort study. Lancet Respir Med. 2022 Sep;10(9):863–76.

Supporting information

S1 Table. Full regression analysis results.

Figure 1

Click here to access/download;Figure;Fig1.tif

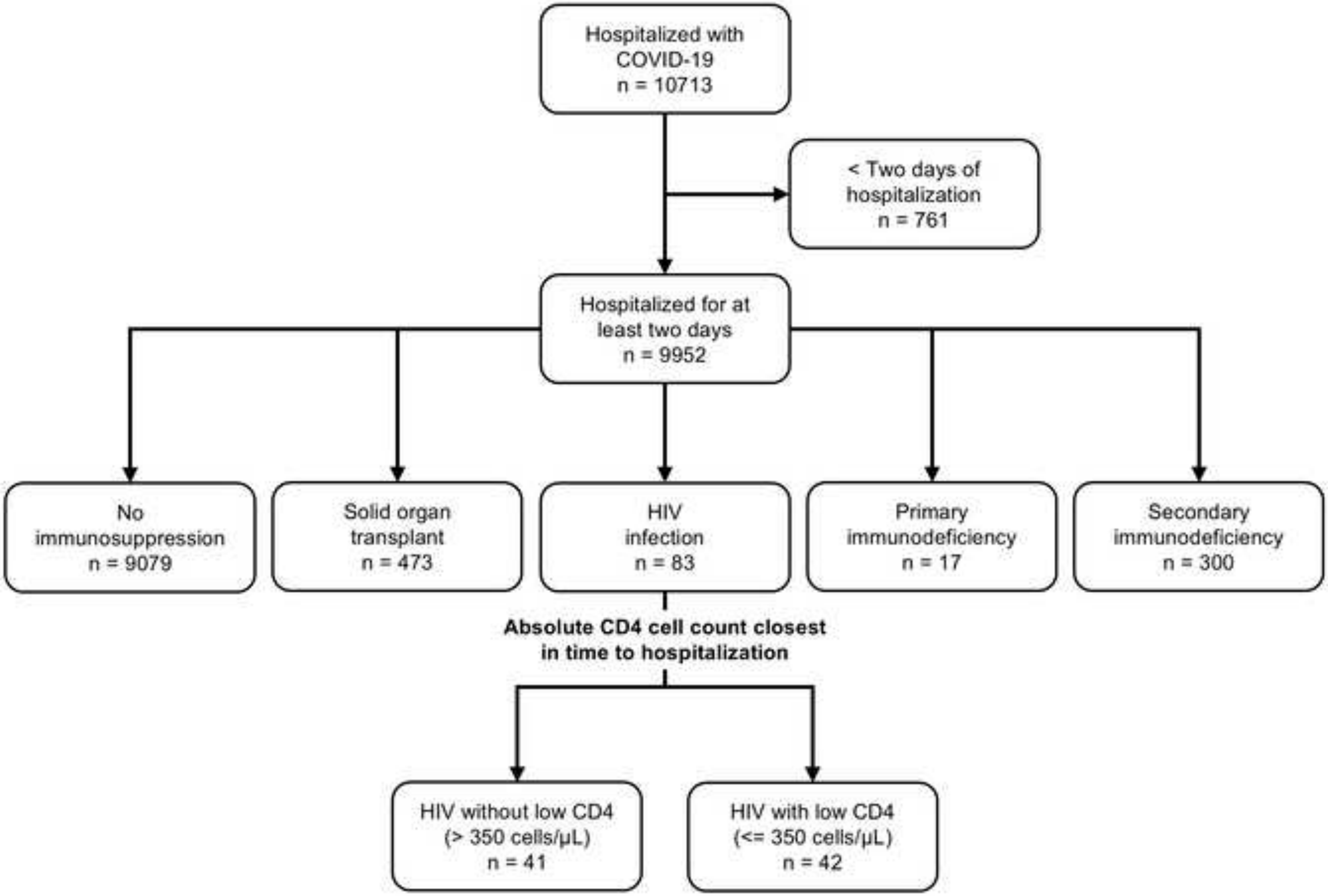

Supporting Table 1

Click here to access/download
Supporting Information
S1_Table.docx

Click here to access/download
Other
Correspondence_for_STU00217318.pdf

Click here to access/download
Other
NU.IRB.STU00217318 Continued Approval.pdf
