# Supplementary material for: Immunosuppression variably impacts outcomes for patients hospitalized with COVID-19: A retrospective cohort study
Source: PLoS One. 2025 Aug 8;20(8):e0330110. doi: 10.1371/journal.pone.0330110 (PMC12334029; doi:10.1371/journal.pone.0330110)
Supplement: Attachment — Submitted filename: Response to Reviewers.docx [file pone.0330110.s009.docx]

Response to Reviewers and Academic Editor

Thank you to everyone for their thoughtful comments and edits that have helped further strengthen this manuscript.

The original comments from the Reviewers and Academic Editor are in *italics*, and my responses are below each comment in regular text. When appropriate, line numbers/sections to refer to and specific changes made are available in blue text under each response. Important deletions are marked as “DELETED.” Line numbers are referring to those in “Revised Manuscript with Track Changes.”

The comments from Reviewers 1-3 are addressed first, and the comments from the Academic Editor are addressed afterward. Additional Journal Requirements are addressed last.

**Response to Reviewer 1**

*Comment 1: “The only part that might be considered to be omitted from the manuscript is data about the patients' insurance as this does not share any important information in this research.”*

We thank the reviewer for bringing up the fact that the insurance information does not add to this research. We have omitted the data about patients’ insurance status from Table 1 as suggested.

See Table 1 (line 384).

**Response to Reviewer 2**

*Comment 1: “Results section: Hospitalization outcomes; it is not clear whether the hospitalization mentioned here includes ICU hospitalization. This has also to be clarified in table 2 since there is another table i.e. Table 3 with ICU admissions.”*

We appreciate the request for clarification on these outcomes. All patients included in this study were hospitalized, but not all patients were also admitted to the ICU. Hospitalization length is defined as the total amount of time a patient spent in the hospital, which would also include the time they spent in the ICU if they were admitted to the ICU. The language in the Results and Methods section has been updated to make this clearer and strengthen the manuscript. The tables have also been updated to make it clear that ICU admission is a distinct outcome that occurs at different rates in the different groups.

Line 266: “For patients who were also admitted to the ICU, hospitalization length includes time spent in the ICU”

Line 426: “Our study only included hospitalized patients, and many of these patients were also admitted to the ICU while hospitalized”

See Table 3 (line 481).

*Comment 2: “Discussion: Check paragraph 4, While the authors mention that they did not observe any significant difference in length of stay in ICU for PWH with low CD4 counts, table 3 and the results section show otherwise.”*

We thank the reviewer for bringing up a potential discrepancy in our discussion. We double checked our reporting and discussion of ICU length of stay in PWH with low CD4 counts. The adjusted p-value for ICU length of stay for this group was 0.36 and therefore not significant, and this is reflected in Table 5. This is reported in our Results section where we state “PWH with low CD4 had no significant differences in hospitalization length or ICU length of stay.” This is also consistent with our Discussion section where we state “However, while this study found that PWH with low CD4 cell count were hospitalized for significantly longer, we did not observe any significant differences in hospitalization length, oxygenation requirements, or length of ICU stay compared to patients without immunosuppression.” Therefore, the Discussion is consistent with the Results and Table 3.

See Table 5 (line 552)

Line 543: “PWH with low CD4 had no significant differences in hospitalization length or ICU length of stay”

Line 592: “However, while this study found that PWH with low CD4 cell count were hospitalized for significantly longer, we did not observe any significant differences in hospitalization length, oxygenation requirements, or length of ICU stay compared to patients without immunosuppression”

**Response to Reviewer 3**

*Comment 1: “Please consider non-parametric methods: log transformation in the study is not suitable for two reasons. Firstly, the summary statistics on length of stay are presented as mean(SD) which is not appropriate when the data is skewed; consider summary stats for non-normal data; secondly, the log transformation of LOS in the regression analysis was motivated by need to have normality of the distribution of residuals. This would require back-transformation of estimates from “log” residual model . It is not clear how this was accomplished in the is study.”*

We appreciate the reviewer for considering the best methods to present summary stats and for asking us to elaborate on our analyses. We agree that mean(SD) may not be appropriate when the data is skewed, and we have accordingly changed all the summary stats to reflect median and interquartile range (IQR) instead as these are the appropriate summary statistics to use when the normality of data is not explicitly validated. We have also strengthened the manuscript by adding explanation in the methods section of how back-transformation of estimates from the log residual model were accomplished using exponentiation via the emmeans R package.

See Table 1 (line 384).

See Table 5 (line 552).

Line 315: “For hospitalization length, the logarithm transform of the data was used to achieve normality of the residuals and back-transformation of estimates from the log residual model were produced using exponentiation via the emmeans R package”

*Comment 2: “please state if there were the competing risks to compute cumulative incidence of ICU admission and all-cause mortality censored at discharge”*

We thank the reviewer for bringing this into consideration. Because we are using retrospective EHR data, which is irregularly sampled, we cannot consistently assess competing risks across all participants. As the reviewer suggests, this is an important limitation to mention. We have added a statement in the Methods section that competing risks were not used when analyzing the incidence of ICU admission and all-cause mortality. We have also mentioned this as a limitation in the Discussion section and added further context on why it is a limitation of our data source.

Line 305: “We did not apply competing risks analysis when assessing these binary variables”

Line 673: “In addition, given the irregularity of EHR data collection, another limitation is that it is difficult to consistently assess competing risks across all participants, so we did not use competing risks analysis when assessing the cumulative incidence of the binary outcomes. Thus, these outcomes may be vulnerable to effects on competing risks”

*Comment 3: “Clarify the part “and all-cause mortality censored at discharge.”*

We appreciate the reviewer’s request for clarification. We had meant that patients were classified as surviving at time points that occurred after they were discharged alive. Now that we have simplified our outcomes based on other comments/feedback to look at overall in-hospital mortality and not mortality at different time points, this statement is no longer necessary, so we have removed it. The assessment of mortality should be much clearer overall now.

See Table 2 (line 419).

Line 290: DELETED “Fourteen and 28-day cumulative incidence of ICU admission and all-cause mortality censored at discharge were also assessed”

*Comment 4: “Would the authors provide more information how influential values and multicollinearity were assessed?”*

We thank the reviewer for finding ways to strengthen the Methods of our manuscript. Influential values were assessed by isolating any observations with a standardized residual greater than three and confirming that removal of these observations did not impact the results. Multicollinearity was assessed using the variance inflation factor. This information has been added to the Methods section, further strengthening the manuscript.

Line 306: “For all logistic regression analyses, influential values were assessed by isolating any observations with a standardized residual greater than three and performing sensitivity analyses. The variance inflation factor was used to confirm that there was no multicollinearity observed across all the analyses”

*Comment 5: “Age of patients is presented as mean(SD). This is recommended when data is assumed to be normally distributed, however, there does not seem to be validation of this assumption. The authors should provide clarification support for the choice of mean(SD) for age.”*

We appreciate the reviewer’s thorough perusal of our summary stats. We have changed all the summary stats (including that for age) to reflect median and interquartile range (IQR) instead of mean(SD) as these are the appropriate summary statistics to use when the normality of data is not explicitly validated.

See Table 1 (line 384).

*Comment 6: “Hospitalization length was presented as mean(SD) , while the multivariate regression analysis also recognized that it is non-normality of the residuals by applying log transformation. This would require back-transformation of estimates from “log” residual model . Further, the adequacy of transformation was not discussed. It is not clear how this was accomplished in the is study.”*

We appreciate the reviewer’s thorough perusal of our summary stats and gaps in our log residual model explanation. We have changed all the summary stats (including that for hospitalization length) to reflect median and interquartile range (IQR) instead of mean(SD) as these are the appropriate summary statistics to use when the normality of data is not explicitly validated. We also added explanation in the methods section of how back-transformation of estimates from the log residual model were accomplished using exponentiation via the emmeans R package.

See Table 5 (line 552).

Line 315: “For hospitalization length, the logarithm transform of the data was used to achieve normality of the residuals and back-transformation of estimates from the log residual model were produced using exponentiation via the emmeans R package”

*Comment 7: “ICU admission length was presented as mean(SD) , while the multivariate regression analysis also recognized that it is non-normality of the residuals by applying log transformation. This would require back-transformation of estimates from “log” residual model . Further, the adequacy of transformation was not discussed. It is not clear how this was accomplished in the is study.”*

We appreciate the reviewer’s thorough perusal of our summary stats. While log transformation was applied for hospitalization length, it was not needed for ICU admission length. Nonetheless, we have changed all the summary stats (including that for ICU admission length) to reflect median and interquartile range (IQR) instead of mean(SD) as these are the appropriate summary statistics to use when the normality of data is not explicitly validated.

See Table 5 (line 552).

*Comment 8: “While most of the methodology was described to facilitate reproducibility, additional details on how assumptions on normality of data, appropriateness/adequacy of log-transformed length of stay models using multivariate regression would enhance the ability of other research to reproduce the study. Cumulative incidence of binary outcomes at 14 day and 28 day timepoint may be vulnerable to effects on competing risks. This was not addressed, and neither was it discussed as a limitation.”*

We sincerely appreciate the reviewer’s detailed suggestions to improve discussion of our methodology. As suggested, we have removed any assumptions on normality of data (opting for non-parametric summary statistics of median and IQR), and we have further explicated the use of a log-transformed model for hospital length of stay and the appropriate back-transformations that were performed. Regarding the feedback that binary outcomes at 14 day and 28 day timepoints may be vulnerable to effects on competing risks, we thought about this carefully and decided that it would be more useful to simply focus on the outcomes during all of hospitalization instead of trying to assess outcomes at these time points. This would also be less vulnerable to competing risks. Thus, we have changed our binary outcomes to reflect occurrence during all of hospitalization. We have also addressed the fact that we did not use competing risks analysis for our binary outcomes, and we now discuss this as a limitation in the Discussion section.

See Table 1 (line 384).

See Table 5 (line 552).

Line 315: “For hospitalization length, the logarithm transform of the data was used to achieve normality of the residuals and back-transformation of estimates from the log residual model were produced using exponentiation via the emmeans R package”

Line 259: “The primary outcome was in-hospital mortality. Secondary outcomes included hospitalization length, oxygenation requirements, and ICU admission/length of stay”

See Table 2 (line 419).

See Table 3 (line 481).

See Table 4 (line 527).

Line 305: “We did not apply competing risks analysis when assessing these binary variables”

Line 673: “In addition, given the irregularity of EHR data collection, another limitation is that it is difficult to consistently assess competing risks across all participants, so we did not use competing risks analysis when assessing the cumulative incidence of the binary outcomes. Thus, these outcomes may be vulnerable to effects on competing risks”

**Response to Academic Editor**

*Comment 1: I have revised this title like this because their immunocompromising status is a life-long condition, not just a "pre-COVID" condition. Furthermore, solid organ transplant is not an immunosuppressive condition, but patients who underwent solid organ transplantation are considered individuals with an immunocompromising status mainly because of their concomitant treatment with immunosuppressants. Because "hospitalization" for COVID-19 is a study outcome, the word "hospitalized" needs to be removed from the manuscript's title.*

We thank the editor for their suggestions on improving the title. To provide clarification, the intent of this study was to assess post-hospitalization outcomes in patients who were hospitalized with COVID-19, and all 9952 patients in this study were hospitalized with COVID-19. Thus, hospitalization is not a study outcome, but rather one of the inclusion criteria for this study. We have clarified this in the Methods section description of inclusion criteria, and we are therefore inclined to keep the word “hospitalized” in the manuscript title as it describes an important characteristic of the population in this study. As for the distinction that immunosuppression is not just a condition, we agree with your assessment and have updated the title accordingly.

Line 1: “Immunosuppression variably impacts outcomes for patients hospitalized with COVID-19: A retrospective cohort study”

*Comment 2: If the NM health system database is representative of a US population, you could say "retrospective population-based cohort study"*

We appreciate the editor considering this as a possibility. However, as we mention in the Discussion section, one limitation of our study is that NM is an academic health system with more complex, sicker, and better insured patients compared to community-based hospital systems, meaning it may not be fully representative of the general population. Therefore, we think it is best to keep this as “retrospective cohort study.”

Line 677: “Finally, our study is limited to a single health system and therefore may not be representative of the general population. This is especially important given NM is an academic health system with more complex, sicker, and better insured patients compared to community-based hospital systems”

Line 1: “Immunosuppression variably impacts outcomes for patients hospitalized with COVID-19: A retrospective cohort study”

*Comment 3: Consider numbering lines of the manuscript to ease its assessment.*

We have numbered the lines of the manuscript as suggested.

*Comment 4: Maximum word count = 300*

We have kept the Abstract to less than 300 words as instructed.

*Comment 5: I am happy that you use the word "adult" here, but it should also be frequently used throughout the manuscript so that readers can clearly understand that exposed study participants are adult immunocompromised individuals.*

We have added the word “adult” throughout the manuscript as appropriate.

See lines 153, 154, 331, 337, 338, 339, 558, 559, 561, 697.

*Comment 6: To keep consistency with my comments below, you need not informing readers about the subgroups of immunocompromised individuals included in this study at this level of the manuscript.*

We appreciate the editor’s consideration of how the abstract could be strengthened. We have read through your comments about having an exposure (all patients with immunosuppressive status) and non-exposure group (all patients without immunosuppressive status), and we have added this grouping/comparison as we agree it would be useful to include. We have added this to the abstract as well. However, one of the main intents of our paper was to separately present outcomes for different reasons for immunosuppression. In the immunosuppression field, we believe readers are very interested in how patients with specific forms of immunosuppression fare rather than how a combined group with various forms of immunosuppression fares. This is because the various forms of immunosuppression are very different both clinically and biologically. Thus, while we have followed your suggestion to remove the details of the subgroups in the background portion of the abstract, we still believe it should be mentioned in other parts of the abstract (discussed in further comment responses below).

Line 40: “Adults with immunosuppression are more likely to develop severe COVID-19 than adults without immunosuppression. Less is known about differences in outcomes for adults with immunosuppression who are hospitalized with COVID-19”

*Comment 7: What do you term "secondary immunodeficiency? Someone could consider HIV to be a secondary immunodeficiency because its cause is known. So, in the "Exposure and outcomes" sub-section of the Methods section in the full-text, consider specifying the conditions that were considered as being part of the secondary immunodeficiency group in this cohort study and as mentioned below, specify why you selected those conditions out of the whole secondary immunodeficiency group.*

We thank the editor for asking us to define this better. We use the term “secondary immunodeficiency” to refer to patients taking immunosuppressive medications for autoimmune disease and patients who have received stem cell transplants. This is now clarified in the “Exposure and outcomes” sub-section of the Methods, and we have also explained why we selected these conditions.

Line 245: “SI (includes patients taking immunosuppressive medication for autoimmune disease and patients who have received hematopoietic stem cell transplants). We defined SI in this way to capture common reasons for immunosuppression which we noted in our cohort that did not fall into the other groups we identified. This also aligned with representation of SI in the literature”

*Comment 8: I suggest "saying "subjects without the immunocompromising status" throughout the manuscript because like that you emphasize on the statuses "exposed and "non exposed" which are commonly known in cohort studies. Alternatively, instead of keep saying "immunocompromised and non-immunocompromised individuals, you could just say "the exposed" and the "non-exposed" throughout the manuscript and even in the abstract. Going through the "Exposure and outcomes" sub-section of the Methods section, readers will clearly understand which group of participants is the exposed and which group is the non-exposed.*

We appreciate the editor’s suggestions to be consistent throughout the manuscript with how patients are referred to. We agree that saying “without immunosuppression” is better than saying immunocompetent, and we have updated this language throughout the manuscript. We also considered the alternative suggestion to say exposed and non-exposed throughout the manuscript, and have used this language in the appropriate places throughout the manuscript. However, we still think there are places where the first alternative to say with and without immunosuppression makes the manuscript read more clearly and have also updated that language accordingly.

Exposure and non-exposure: see lines 194, 195, 203, 238, 239, 299, 301, 314, 315, 352, 353, 354, 359, 364, 384 (Table 1), 389, 390, 415, 416, 417, 419 (Table 2), 422, 481 (Table 3), 484, 488, 527 (Table 4), 529, 552 (Table 5), 554, 571, 572, 659.

With immunosuppression: see lines 40, 50, 54, 60, 61, 77, 144, 239, 352, 384 (Table 1), 389, 416, 428, 491, 497, 504, 533, 534, 538, 571, 574, 697, 702, 703, 706, 880, 881.

Without immunosuppression: see lines 42, 55, 62, 66, 78, 82, 154, 194, 238, 239, 352, 384 (Table 1), 390, 401, 402, 403, 406, 415, 423, 430, 435, 436, 437, 484, 485, 487, 488, 490, 491, 497, 500, 501, 503, 505, 509, 529, 535, 538, 539, 542, 543, 554, 559, 572, 576, 595, 596, 598, 601, 605, 606, 608, 698, 880.

*Comment 9: As mentioned in my comments for the results sub-section of the abstract, do not detail which subgroups were included in the exposed study population. It will be available in the full-text for interested readers.*

We thank the editor for reminding us that details of the sub-groups will be available in the full text and that this may be redundant to have here. We have removed the details of the subgroups included in the exposed study population at the Methods level of the abstract.

Line 50: DELETED “solid organ transplant (n = 473), HIV without (n = 41) and with low CD4 cell count (n = 42), primary immunodeficiency (n = 17), and secondary immunodeficiency (n = 300)”

*Comment 10: Provide results of the whole "exposed population" compared to the non-exposed population. Regarding results of subgroups of the exposed population, only highlight results of the subgroup who had the poorest outcomes. This is a strategy that could help you gain more readers accessing the full-text.*

We appreciate the editor’s suggestions for helping us gain more readers accessing the full text. As suggested, we have added in results of the whole exposed population compared to the non-exposed population. We have also edited this section as suggested to highlight the results of the subgroup who had the poorest outcomes (solid organ transplant). However, we feel it is important to highlight not only the sub-groups who had the poorest outcomes, but also to highlight the sub-groups which performed fairly well (HIV with normal CD4 cell count). We actually believe this would be very interesting to readers because immunosuppression is commonly expected to lead to poorer outcomes, so results against this are always very intriguing. Therefore, while we have simplified the abstract as suggested and focused on the whole exposed population compared to the whole non-exposed population, as well as the group with the poorest outcomes, we left in some additional subgroup details (within the word limit) to make the abstract more enticing.

See Results section of Abstract (line 54).

*Comment 11: Same comment as for the results sub-section of the abstract.*

Please refer to our responses above about the similar set of comments.

See Results section of Abstract (line 54).

*Comment 12: Keywords should be added and retrieved from the manuscript's title and the abstract.*

The keywords have been added.

Line 83: “Keywords: COVID-19; immunosuppression; hospitalization; solid organ transplant; HIV/AIDS; primary immunodeficiency; secondary immunodeficiency”

*Comment 13: Consider revising the introduction. COVID-19 is well known even in the general public. So, I do not think you need to narrate on the phases of COVID-19 outbreak in this manuscript. I suggest a 3-paragraph text on what is known about the epidemiological relationship between immunosuppression and COVID-19, what is unknown about that epidemiological relationship, and what is the aim of this study as well as how it will help fill the gap in knowledge. Along this line, I recommend the authors go through the systematic reviews and meta-analyses (e.g., refs 5, 6 and the others mentioned below at the level of references) and check the flaws of previous primary studies and recommendations made by the authors of those systematic reviews and meta-analyses for improving research on this domain (of epidemiological link between immunosuppression and COVID-19). Furthermore, did those systematic reviews and meta-analyses include many studies? Many (population-based) cohorts? Even if they included many cohorts, what was the sample size of those cohorts? Were there many studies coming from the United States? Those are potential gaps of knowledge that would be expected to be mentioned in the second paragraph of the introduction.*

We thank the editor for their suggestions on how the introduction can be revised. We have followed the suggestions and adopted a clearer and more succinct 3-paragraph format that describes what is known, describes what is not known/gaps/flaws in the previous literature, and then describes the aim of this study and how it will fill these gaps.

Line 86: DELETED “Severity of coronavirus disease 2019… nearly 1.9 million deaths in the United States alone”

See Introduction (line 85)

*Comment 14: Consider writing this manuscript based on the STROBE statement guideline: see https://doi.org/10.1371/journal.pmed.0040297. Accordingly, exactly at this level of the manuscript, state that you report this article based on STROBE guidelines and upload a STROBE checklist as supplemental material to demonstrate how you applied those guidelines.*

We have added a statement that we report this article based on STROBE guidelines and uploaded a STROBE checklist as supplemental material to demonstrate how we applied those guidelines.

Line 161: “We report this article based on STROBE guidelines”

See S1 File (line 889).

*Comment 15: This should be the last sub-section of the Methods section. Also consider mentioning whether you abided by rules from the Declaration of Helsinki regarding human patient enrollment in studies: see https://doi.org/10.1038/s41591-024-03433-5 for example.*

We thank the editor for the suggestions on improving the organization of our Methods section. The Ethics statement section has been moved to the end of the Methods section. Our study was approved by the Northwestern University Institutional Review Board, which only approves studies using human subjects data that follow the highest ethical research guidelines, including such rules as found in the Declaration of Helsinki. We have made this clear in the Ethics statement section.

See Ethics statement section of Methods (line 325).

Line 326: “This was a retrospective study performed under a waiver of consent. This study was approved by the Northwestern University Institutional Review Board (STU00217318), which operates under the principles of the Declaration of Helsinki.”

*Comment 16: This will become the first sub-section of the Methods section. I suggest replacing "setting" with "data source" because you collected data retrospectively in a database.*

We appreciate the suggestion to say “data source” instead of “setting,” and we have updated the title of this sub-section accordingly.

See Study design and data sources section of Methods (line 165).

*Comment 17: consider deleting this here because it refers to a setting. Start by reporting the study design in the first sentence of the sub-section. The study design is: "cohort study". Retrospective refers to the timeline of data collection. Accordingly, the word "retrospective" should be moved to the "Data collection" sub-section. After clearly stating the study design, the following sentences should clearly describe the data source. Yes, data are coming from the Northwestern Medicine Enterprise Data Warehouse, but can you describe the NM health system database more comprehensively (though briefly) as authors usually do with other US databases such as NHANES (https://doi.org/10.1038/sdata.2016.96)? Is that database representative of the US database (such that you can say your study is a population-based study?)*

We thank the editor for their suggestions on making the study design section clearer. The first sentence of the sub-section now clearly reports that this is a cohort study, and the word “retrospective” has been moved to the data collection sub-section as suggested. We have also described the NM health system database more comprehensively and discussed that it is not representative of a US database.

Line 166: “We performed a cohort study utilizing data from the Northwestern Medicine (NM) Enterprise Data Warehouse (NMEDW)”

Line 167: “The NMEDW is the clinical research database of NM, a large healthcare system providing inpatient, outpatient, and specialty care throughout Chicago and Northern Illinois. The NMEDW contains data, including diagnosis (ICD-9 and ICD-10 codes), hospitalization, treatment, laboratory, and demographic information for over 10 million patients who have received care at NM hospitals and clinics”

Line 190: “All data was extracted retrospectively”

Line 677: “Finally, our study is limited to a single health system and therefore may not be representative of the general population. This is especially important given NM is an academic health system with more complex, sicker, and better insured patients compared to community-based hospital systems”

*Comment 18: Full-spelling of this word is needed here.*

The full spelling of EHR (electronic health record) has been added upon its first use in the Methods section.

Line 232: “Given the missingness inherent in electronic health record (EHR) data”

*Comment 19: Yes, this should be the second sub-section of the Methods section.*

We thank the editor for this confirmation.

*Comment 20: This sub-section should be revised to clearly delineate inclusion and exclusion criteria. I propose sentences like this to enumerate eligibility criteria: "The inclusion criteria were...The exclusion criteria were: ....Before addressing eligibility criteria, the authors should address the sampling issue*

We appreciate the editor’s suggestions to improve the clarity of our inclusion and exclusion criteria. We have revised this sub-section accordingly to clearly delineate the eligibility criteria. We have also commented on the sampling issue as suggested.

Line 175: “We sampled patient data from the NMEDW. The inclusion criteria were receiving a positive COVID-19 laboratory test (positive polymerase chain reaction on nasopharyngeal or lower respiratory tract specimens, positive SARS-CoV-2 antigenic test) between 03/01/2020 and 05/31/2022, being hospitalized in the period encompassing one day before to seven days after the laboratory test result, and being at least 18 years of age at the time of hospitalization. The exclusion criteria were not being hospitalized, testing positive for COVID-19 more than one day after hospitalization, and being hospitalized for less than two days”

*Comment 21: There is a need for consistency with the terminology used for the data source throughout the manuscript: NM or NM health system database?*

We thank the editor for recognizing that we need to be more consistent with our terminology. To clarify, the NM Enterprise Data Warehouse (NMEDW) contains all the electronic health record data for patients who have received care within the NM health system (comprised of multiple hospitals). As suggested above, we have added more details about the NM health system database. As suggested here, we have made the data source terminology throughout the manuscript consistent. It is now referred to as the NMEDW (which is where we pull the data from) throughout the manuscript.

See lines 167, 171, 175, 190, 228.

*Comment 22: Yes, this sub-section can be the third one of the Methods section. However, you cannot merge the "Data collection" sub-section with the definition of terms. Consider reducing the length of this "Data collection" sub-section. Focus on the description of the timeline of data collection: retrospective, people who collected data and whether they were trained for data collection before undertaking the task or not, where data were recorded (electronic questionnaires such as REDCap questionnaires or print questionnaires, the types of data collected. Regarding the types of data collected, focus on data about the exposure (which is the "immunosuppression status") and data necessary to assess the study outcomes (hospitalization, ICU admission, respiratory outcome, all-cause mortality and COVID-19-specific mortality. While reporting on the data collection process, highlight whether you made every effort to minimize biases that can be due to data collection, especially since you retrospectively collected data, meaning that there is a high risk for recall bias.)*

We appreciate the editor’s suggestions to keep the Data collection sub-section distinct from definitions and shorten it appropriately. We have made the Data collection sub-section distinct and significantly clearer/shorter, focusing on data about exposure and outcomes as suggested. We have also added information about the people who collected data and efforts to minimize bias in data collection as suggested. We want to note that since this is an EHR-based study where we identify everyone we can from the EHR, there is less control over and impact of recall/selection bias. In particular, our inclusion criteria were as broad as possible to inherently minimize this type of bias. All of this is discussed extensively now in the Methods and Discussion.

See Data collection section of Methods (line 189).

Line 190: “All data was extracted retrospectively from the NMEDW by power users of the NMEDW trained for data collection”

Line 230: “Bias during data collection was minimized by defining our study population broadly and extracting all relevant data available. Given the missingness inherent in electronic health record (EHR) data due to its real-world nature, we also chose data types known to have low missingness (i.e. admission dates, in-hospital mortality) to capture study outcomes”

*Comment 23: This is for example an information needed when describing the NM health system database: that diagnosis is made using ICD codes, with 9th and 10th revisions used for this study. It contains information about patients morbidities, treatments, hospitalizations, health insurance...*

We appreciate the request for more information. We have made sure to include this information (regarding what is included in the database) in the added description of the NM health system database as discussed above.

Line 171: “The NMEDW contains data, including diagnosis (ICD-9 and ICD-10 codes), hospitalization, treatment, laboratory, and demographic information for over 10 million patients who have received care at NM hospitals and clinics”

*Comment 24: This is an information for the "Exposure and outcome" sub-section which is required in a cohort study like this one.*

This information about patient assignment to HIV subgroups has been moved to the Exposure and outcomes sub-section as suggested. The information about extraction of CD4 data is also still available in the Data collection sub-section as appropriate.

Line 240: “further divided into the following subgroups: SOT (includes kidney transplant, liver transplant, heart transplant, or lung transplant), HIV without low CD4 (absolute CD4 cell count > 350 cells/µL), HIV with low CD4 (absolute CD4 cell count <= 350 cells/µL)”

Line 201: “Absolute CD4 cell counts closest to the time of hospitalization including the period encompassing seven days after hospitalization were also extracted for exposure subgroup assignment”

*Comment 25: I suggest avoiding this terminology in this article because the char review is a study design (https://doi.org/10.3352/jeehp.2013.10.12).*

We thank the editor for recognizing this potentially confusing terminology. We have updated this to say “manually adjudicated” instead of “performed chart review” throughout the manuscript.

Line 252: “All patients assigned to an immunosuppression subgroup were manually adjudicated”

Line 334: “Of these, 1123 patients were manually adjudicated”

Line 652: “In addition, while we manually adjudicated”

*Comment 26: Furthermore, this sentence repeats the information already provided above.*

We appreciate the editor’s concerns that this sentence about adjudicating patients with immunosuppression is redundant. However, we believe this is an important point to include about how a patients’ immunosuppression was confirmed, and we believe this adds information that is not already stated above (as we only discuss identification with diagnosis codes above). Diagnosis codes are not always 100% reliable, and this is a known problem in the field of electronic health record-based phenotyping. Therefore, some amount of manual adjudication is usually expected to ensure that patients have been accurately classified. In this study, we went even further and adjudicated all patients who were identified as with immunosuppression based on diagnosis codes. Therefore, we think this point is important to include as it shows how our study is even stronger in that there was researcher confirmation of each patient with immunosuppression and the group they should belong to. Note that this sentence has been moved with the above sentences to the Exposure and outcomes sub-section.

Line 252: “All patients assigned to an immunosuppression subgroup were manually adjudicated to accurately classify categorization of their immunosuppression”

*Comment 27: For the "Exposure and outcome" sub-section.*

We have moved this text about immunosuppression category reassignment based on manual adjudication to the “Exposure and outcomes” sub-section as suggested.

Line 253: “Patients found to have a different immunosuppression subgroup than expected based on ICD coding were reassigned to the appropriate group”

*Comment 28: As mentioned above, no need to maintain this sentence once you have well described the NM health system database.*

We have simplified this information about the diabetes covariate as suggested now that the NM health system database is well-described above.

Line 215: DELETED “ICD-9 and ICD-10 diagnosis codes were used to define whether patients had a history of diabetes”

Line 224: “To assess study covariates and sample demographics, age at admission, sex assigned at birth, race, ethnicity, most recent body mass index (BMI) measurement, diagnosis codes for diabetes, and in-hospital use of remdesivir and Paxlovid were extracted”

*Comment 29: Did you also extract information about COVID-19-specific mortality? This is an important outcome data for this study.*

We appreciate this important question from the editor. Unfortunately, the NM health system database does not contain information on cause-of-death-specific mortality. This is why we chose in-hospital all-cause mortality as the next best thing.

*Comment 30: This is an information for the statistical analysis sub-section.*

Along with the information mentioned in the comment below about variant eras in our study, this information about variant era assignment has been summarized and moved to the Exposure and outcomes sub-section. It is also explained as appropriate in the Statistical analysis sub-section.

Line 301: “We chose to adjust for these covariates because age, diabetes, and obesity have all been identified as the strongest risk factors for death in patients hospitalized with COVID-19, and because of changes in pandemic landscape over time with the rise and fall of different VOCs and the introduction of new treatment options”

*Comment 31: This is an information for the "Exposure and outcome" sub-section.*

Along with the information mentioned in the comment above, this information has been summarized and moved to the Exposure and outcomes sub-section.

Line 278: “Dates of hospital admission were also used to assign SARS-CoV-2 variant era with categorization into three groups based on calendar time of when a specific variant of concern (VOC) accounted for greater than 50% of circulating viruses determined per sequencing surveillance. The eras falling within our study timeline were defined as: Pre-Delta (ancestral and early variants of concern including alpha and gamma, 03/01/2020 to 06/30/2021), Delta (07/01/2021 to 12/31/2021), and Omicron (01/01/2022 to 05/31/2022)”

*Comment 32: Is this information necessary in this manuscript? Due to space constraints, only mention what is necessary in this article.*

We appreciate the editor questioning whether this information about date of first data access and author access to information is necessary. We initially did not have this information in the manuscript, but later added it based on the guidelines about reporting data access on the PLOS One website. If the editor believes this information is unnecessary, we are happy to remove it as it is not necessary to the article.

Line 235: DELETED “All data were first accessed on 05/21/2023 for research purposes. Authors had access to information that could identify individual participants during and after data collection”

*Comment 33: As mentioned above, this is a very important sub-section of cohort studies, including this one. In this sub-section, the first part should address the exposure which is the "immunosuppression status" because you are assessing whether the immunosuppression status influences COVID-19 infection outcomes. While describing the health conditions that were considered within the "immunosuppressive status", elaborate on why you selected HIV, solid organ transplant, primary immunodeficiency among the multiple immunocompromising states known. Also explain why you focused on the outcomes you chose to assess, with proper characterization of each outcome: were there primary and secondary outcomes? This should also be specified. Accordingly, the duration of follow-up for each observation should be clarified here. Because of the "Exposure and outcomes" sub-section you need not adding a "Definitions" sub-section.*

We thank the editor for all the comments, including this one, regarding the important addition of an Exposure and outcomes sub-section. We have added this sub-section and followed suggestions to address exposure in the first part, explain why exposure subgroups were chosen, further elaborate on outcomes, and clarify the duration of follow-up. We have accordingly removed the Definitions sub-section as suggested.

See Exposure and outcomes sub-section of Methods (line 237).

Line 189: DELETED “Definitions”

*Comment 34: Indeed, the "Statistical analysis" sub-section should follow the "Exposure and outcomes" sub-section. While implementing reviewer 3's recommendations into the "Statistical analysis" sub-section, focus on describing the software used for statistical analysis and whether you had to transfer data from one software to another, the statistical tests used and the corresponding data analyses (including details about model fitting and model validation). Also state how data were deemed to be statistically significant and how results are reported in the manuscripts including tables, charts, figures, summary statistic measures and effect sizes. Although loss to follow up is not a big issue in retrospective cohorts as in prospective cohort studies, consider explaining how you accounted for missed observation (if any) during the analysis of study outcomes. All data segregations performed should also be reported here.*

We thank the editor for their suggestions about the Statistical analysis section. In line with this comment and others regarding this section from both the editor and the reviewers, we have made this its own section and extensively revised it accordingly. The software used for statistical analysis is emphasized, and the statistical tests/data analyses used are described in detail. We also stated our threshold for statistical significance, and explicitly described how results (including summary statistic measures and effect sizes) are reported. Finally, we added a statement that there we no missing observations.

See Statistical analysis sub-section of Methods (line 296).

*Comment 35: Based on my comment just above, this text under the statistical analysis sub-section should be revised.*

This text about the continuous variables and linear regression analyses has been revised accordingly.

Line 313: “Multivariate linear regression analyses adjusting for the same covariates were performed in R to assess the effect of exposure on hospitalization length and ICU length of stay. The effect of exposure subgroup was assessed in the same way. For hospitalization length, the logarithm transform of the data was used to achieve normality of the residuals and back-transformation of estimates from the log residual model were produced using exponentiation via the emmeans R package. Median and interquartile range were used to report summary statistics. Comparison results are reported as unadjusted and adjusted odds ratios with 95% confidence intervals”

*Comment 36: This text needs to be further strengthened consistently with revisions of table 1.*

This text regarding demographic differences between the study groups has been further strengthened consistently with revisions of Table 1.

Line 351: “The median age of the total included cohort was 63 years. Compared to the non-exposure (without immunosuppression) group, the exposure (with immunosuppression) group was significantly younger (median: 59 years exposure, 64 years non-exposure, p-value < 0.001). Of the exposure subgroups, PWH without and with low CD4 cell count had the lowest median ages. Among the included cohort, 47.3% were assigned male and 52.7% were assigned female at birth. The proportion of men was significantly higher in the exposure group (58.7%) compared to the non-exposure group (46.1%, p-value < 0.001). Similar proportions of men to women were seen in patients with PI, but 59.4% and >63.4% of patients with SOT and PWH without low CD4 cell count were men, respectively. There were also significant differences in race, SARS-CoV-2 variant era, BMI, diabetes, and remdesivir use between patients in the non-exposure and exposure groups (p-value < 0.001 for all comparisons)”

See Table 1 (line 384).

*Comment 37: This is good for the supplemental material to give more space to results of the outcome analysis.*

We have moved this flow diagram Figure to the supplemental material, and it is now S1 Fig. It has also been updated to reflect the total exposure and non-exposure groups before splitting into sub-groups.

See S1 Fig (line 877).

*Comment 38: Before data segregation, always present data of the whole exposed group compared to the non-exposed group. Although the use of p-values is highly controversial, I suggest adding a column for p-values to enhance group differences.*

We have added a column for the whole exposed group and a column for p-values as suggested to Table 1.

See Table 1 (line 384).

*Comment 39: Why did you exclude transgender individuals when research should be inclusive as recommended by the world association of medical editors? Justification needed in the "Data collection" sub-section of the methods section.*

We appreciate the editor asking this important question to promote inclusivity. To clarify, we did not exclude anyone based on gender for this study. The only stratification we had available to us from the NM health system database for showing sex distribution for this cohort was sex assigned at birth, which only has two categories (male or female) in the NM health system database. While efforts are being made across the United States to collect this information at a better level of detail, this is unfortunately how it is currently collected in our health system. Therefore, this is the stratification we present, but this does not mean anyone was excluded based on gender or sex. We have added the necessary details/justification to the Data collection sub-section of Methods. It is also worth noting that even if it were possible, specifying the number of transgender individuals in our population might increase the risk of reidentification given that this population is likely to be very small in number.

Line 227: “Demographic variables were stratified to the same level of detail present in the NMEDW (i.e. sex assigned at birth is recorded as either Male or Female”

*Comment 40: Why that ethnic stratification? Please, justify in the "Data collection" sub-section of the Methods section.*

We thank the editor for asking this important question. In the United States, this is how ethnicity is stratified by the Office of Management and Budget of the United States Census Bureau. Thus, this is the standard for how US health systems and academic studies collect, stratify, and present ethnicity information. We understand that this is not a universal representation, but we stratified ethnicity in this way as that is how it is stratified in the NM health system database, and we have no additional information to make different/additional stratifications. We have added the necessary details/justification to the Data collection sub-section of Methods.

Line 227: “Demographic variables were stratified to the same level of detail present in the NMEDW (i.e. sex assigned at birth is recorded as either Male or Female; ethnicity is recorded as either Hispanic or Latino, Not Hispanic or Latino, or Other)”

*Comment 41: Do not forget to mention in the "Statistical analysis" subgroup of the Methods that after the overall analysis, you segregated data by phase of COVID_19 outbreak as well.*

We thank the editor for this reminder. To clarify, phase of COVID-19 outbreak was assigned as a covariate in our models, but we didn’t further segregate all our groups based on this variable. The assignment of phase of COVID-19 outbreak is mentioned as a covariate in the Statistical analysis sub-section of Methods for clarity.

Line 297: “Multivariate logistic regression analyses were performed in R adjusting for age at admission, diabetes, obesity, assigned SARS-CoV-2 variant era, and COVID-19 medication use to assess the effect of exposure on in-hospital mortality, administration of low flow oxygen, non-invasive ventilation, and invasive ventilation, and ICU admission. The effect of exposure subgroup was assessed in the same way. We chose to adjust for these covariates because age, diabetes, and obesity have all been identified as the strongest risk factors for death in patients hospitalized with COVID-19, and because of changes in pandemic landscape over time with the rise and fall of different VOCs and the introduction of new treatment options”

*Comment 42: Consider adding clinical data about COVID-19 at baseline in this table. In a study where you assessed the epidemiological influence of immunosuppression on COVID-19 outcomes, it is important to mention in this table the COVID-19 drug treatments administered to your patients, especially since some immunosuppressive/immunomodulatory drug treatments are also COVID-19 drug treatments, and I suggest considering those COVID-19 drug treatments as covariates in logistic regression analyses. Altogether, it is often important to mention all the covariates in this table.*

We appreciate the suggestion to add data about COVID-19 drug treatments administered to the patients. We have added information about remdesivir and Paxlovid use, which we have available, to Table 1 and described this in the Data collection section. As suggested, we have also added the administration of these treatments as covariates in all of our regression analyses.

See Table 1 (line 384).

Line 171: “The NMEDW contains data, including diagnosis (ICD-9 and ICD-10 codes), hospitalization, **treatment**”

Line 226: “in-hospital use of remdesivir and Paxlovid were extracted”

Line 297: “Multivariate logistic regression analyses were performed in R adjusting for age at admission, diabetes, obesity, assigned SARS-CoV-2 variant era, and COVID-19 medication use”

Controlling for COVID-19 medication use: see lines 57, 392, 431, 495, 537.

*Comment 43: Results of the whole exposed group, before data stratification. When making data stratifications, for all results, do not forget sex-based data stratification.*

We appreciate the editor’s suggestions. We have added a column showing results for the whole exposed group to this table and all the other tables. We have added sex-stratified results to the Supplemental Data.

See Table 1 (line 384).

See Table 2 (line 419).

See Table 3 (line 481).

See Table 4 (line 527).

See Table 5 (line 552).

See S2 Table (line 887).

See S3 Table (line 888).

Line 404: “Similar trends were observed when stratifying results by sex, except that in males, there were no significant differences in mortality for PWH with low CD4 and patients with SI compared to patients without immunosuppression (S2 and S3 Tables)”

Line 437: “Sex stratified results are available in S2 and S3 Tables”

*Comment 44: It is not advisable to merge all those outcomes in a single table. Each outcome is a sub-section of the results section and deserves its tables/figures. Just make sure that no more than 6 figures and tables are included in the main text. the surplus should be included in the supplemental material. If there are incidence outcomes in this article, it should be made known in the "Exposure and outcomes" sub-section of the Methods section. I guess you will have effect magnitude estimates after applying recommendations from reviewer 3. This means tables 2 and 3 will be thoroughly revised and you may add some figures/charts in the manuscript.*

We thank the editor for their suggestions on the tables and figures. We have thoroughly revised how the data is presented so that all the outcomes are not merged in a single table, and that each outcome gets its own table. Effect magnitude estimates have also been presented in the tables as suggested. For our primary outcome of in-hospital mortality, we have also added a figure (Fig 1). Surplus data regarding sex-stratified results is in the Supplemental Tables.

See Fig 1 (line 413).

See Table 1 (line 384).

See Table 2 (line 419).

See Table 3 (line 481).

See Table 4 (line 527).

See Table 5 (line 552).

See S2 Table (line 887).

See S3 Table (line 888).

*Comment 45: Revision needed as recommended by reviewers and in compliance with STROBE guidelines. when addressing study limitations, focus on biases of retrospective cohort studies found here.*

The Discussion section has been revised as recommended by reviewers and to be in compliance with STROBE guidelines. We also added discussion of the biases of retrospective cohort studies when addressing study limitations.

See Discussion (line 556).

Line 655: “Thus, because we only pulled patients who were coded as immunosuppressed in the EHR, we recognize that there may be patients in the without immunosuppression group who should be in one of the immunosuppression subgroups. This is especially true for our SI group, which represents a broad category of immunosuppression. This could potentially bias our non-exposure group toward appearing more severe than they are. However, the magnitude of bias is expected to be small given our comprehensive method of filtering for immunosuppression, the rarity of immunosuppression, and the large size of our cohort. Selection bias is also important to consider in retrospective cohort studies, but there is less control over this form of bias in EHR-based studies and it generally has less impact. In particular, our study broadly identifies as many patients as possible from EHR data. For example, as COVID-19 testing is mandatory for all patients admitted to NM hospitals, selection bias is minimized by making positive COVID-19 testing a key inclusion criterion. Furthermore, by only including patients who were hospitalized for at least 2 days, we avoided including individuals who may not have required hospitalization and kept our study focused on in-hospital outcomes”

*Comment 46: Ponder a proper conclusion section focusing on the main study result and perspectives for future cohort studies on this topic in a short text.*

A conclusion section focusing on the main study result and perspectives for future cohort studies has been added.

See Conclusion (line 696).

Line 697: “Adult patients with immunosuppression experience worse outcomes than adult patients without immunosuppression following hospitalization with COVID-19. Patients with SOT had the poorest outcomes, including higher in-hospital mortality, ICU admission, invasive ventilation, and longer hospital/ICU stay. PWH with low CD4 and patients with SI also had higher in-hospital mortality and ICU admission. These findings underscore the importance of tailored approaches in managing COVID-19 among populations with immunosuppression. Future cohort studies should aim to expand on these results by incorporating multi-health system data to increase sample sizes and improve representativeness. Additionally, investigating long-term outcomes could provide crucial insights into the lasting effects of COVID-19 on individuals with immunosuppression. Such comprehensive research will be invaluable in guiding healthcare providers and public health professionals in their ongoing efforts to protect vulnerable populations during this and future pandemics”

*Comment 47: You also need to insert Declarations (conflict of interests, data availability, author contributions and eventually acknowledgments) here.*

The necessary Declarations have been added.

See Funding statement (line 711).

See Conflicts of interest (line 715).

See Data availability (line 721).

See Author contributions (line 730).

See Acknowledgements (line 742).

*Comment 48: Consider decreasing and updating the reference count. These important references which could replace references 7 and 8 are lacking in this list: Bytyci et al. Immun Inflamm Dis. 2024 Apr;12(4):e1259. https://doi.org/10.1002/iid3.1259 Med Clin (Barc). 2024 Oct 18;163(7):347-359. doi: 10.1016/j.medcli.2024.04.009. Epub 2024 Jun 29. This link may also be helpful for reference updating: https://pubmed.ncbi.nlm.nih.gov/?term=COVID-19+on+immunocompromised+populations&filter=datesearch.y_5&filter=pubt.meta-analysis&filter=pubt.systematicreview*

We thank the editor for their suggestions on a better references section. We have removed the suggested references and incorporated updated references. The reference count is currently 36.

See References (line 755).

*Comment 49: I suggest deleting this reference for example based on my suggestions about how to revise the introduction.*

This CDC reference has been deleted.

See References (line 755).

*Comment 50: Same thing for this reference. It can be omitted.*

This second CDC reference has been deleted.

See References (line 755).

*Comment 51: This reference could be omitted because systematic reviews and meta-analyses have a level of evidence above primary observational studies such as one, and references 5 and 6 are systematic reviews and meta-analyses.*

This Bhaskaran et al. reference has been deleted.

See References (line 755).

*Comment 52: This letter to the editor also needs to be deleted to reduce the reference count.*

This Miyashita et al. reference has been deleted.

See References (line 755).

**Additional Journal Requirements**

*1. Please ensure that your manuscript meets PLOS ONE's style requirements, including those for file naming. The PLOS ONE style templates can be found at*

[*https://journals.plos.org/plosone/s/file?id=wjVg/PLOSOne_formatting_sample_main_body.pdf*](https://urldefense.com/v3/__https:/journals.plos.org/plosone/s/file?id=wjVg*PLOSOne_formatting_sample_main_body.pdf__;Lw!!Dq0X2DkFhyF93HkjWTBQKhk!SPYwApMAsT9eLGNH3w_aiAr7TwkZW6Ki_ojGrNod2TtmBwl3kc-29D1nIVlBcmvjlKusq2-xA7dhK5BUN4C55Ey9$) *and*

[*https://journals.plos.org/plosone/s/file?id=ba62/PLOSOne_formatting_sample_title_authors_affiliations.pdf*](https://track.editorialmanager.com/CL0/https:%2F%2Fjournals.plos.org%2Fplosone%2Fs%2Ffile%3Fid=ba62%2FPLOSOne_formatting_sample_title_authors_affiliations.pdf/1/010f0194498b0ccc-433b39cb-d92f-44b2-a4e8-0606d5a52dbb-000000/nhBVnN0WgQ0U1DDGAkFGoB4Xd_PWuhalw0HnFgdV5hY=192)

I have ensured that my manuscript meets PLOS ONE’s style and file naming requirements.

*2.  We note that the grant information you provided in the ‘Funding Information’ and ‘Financial Disclosure’ sections do not match.
When you resubmit, please ensure that you provide the correct grant numbers for the awards you received for your study in the ‘Funding Information’ section.*

The correct grant number (CO-US-540-6535) is provided in both sections.

Line 712: “TLW received grant number CO-US-540-6535”

Line 716: “TLW received grant number CO-US-540-6535”

*3. Thank you for stating the following financial disclosure:
“TLW received grant number CO-US-540-6535 from Gilead Sciences (*[*https://www.gilead.com/*](https://urldefense.com/v3/__https:/www.gilead.com/__;!!Dq0X2DkFhyF93HkjWTBQKhk!SPYwApMAsT9eLGNH3w_aiAr7TwkZW6Ki_ojGrNod2TtmBwl3kc-29D1nIVlBcmvjlKusq2-xA7dhK5BUN6bWMFEb$)*) for this work.*

*The funders reviewed the final manuscript.”*

*Please state what role the funders took in the study. If the funders had no role, please state: "The funders had no role in study design, data collection and analysis, decision to publish, or preparation of the manuscript."
If this statement is not correct you must amend it as needed.
Please include this amended Role of Funder statement in your cover letter; we will change the online submission form on your behalf.*

The statement has been added in the Conflicts of interest section and in the cover letter.

Line 717: “The funders had no role in study design, data collection and analysis, decision to publish, or preparation of the manuscript. The funders reviewed the final manuscript”

*4. We note that you have indicated that there are restrictions to data sharing for this study. For studies involving human research participant data or other sensitive data, we encourage authors to share de-identified or anonymized data. However, when data cannot be publicly shared for ethical reasons, we allow authors to make their data sets available upon request. For information on unacceptable data access restrictions, please see* [*http://journals.plos.org/plosone/s/data-availability#loc-unacceptable-data-access-restrictions*](https://urldefense.com/v3/__http:/journals.plos.org/plosone/s/data-availability*loc-unacceptable-data-access-restrictions__;Iw!!Dq0X2DkFhyF93HkjWTBQKhk!SPYwApMAsT9eLGNH3w_aiAr7TwkZW6Ki_ojGrNod2TtmBwl3kc-29D1nIVlBcmvjlKusq2-xA7dhK5BUN7K0565E$)*.
Before we proceed with your manuscript, please address the following prompts:
a) If there are ethical or legal restrictions on sharing a de-identified data set, please explain them in detail (e.g., data contain potentially identifying or sensitive patient information, data are owned by a third-party organization, etc.) and who has imposed them (e.g., a Research Ethics Committee or Institutional Review Board, etc.). Please also provide contact information for a data access committee, ethics committee, or other institutional body to which data requests may be sent.
b) If there are no restrictions, please upload the minimal anonymized data set necessary to replicate your study findings to a stable, public repository and provide us with the relevant URLs, DOIs, or accession numbers. Please see* [*http://www.bmj.com/content/340/bmj.c181.long*](https://urldefense.com/v3/__http:/www.bmj.com/content/340/bmj.c181.long__;!!Dq0X2DkFhyF93HkjWTBQKhk!SPYwApMAsT9eLGNH3w_aiAr7TwkZW6Ki_ojGrNod2TtmBwl3kc-29D1nIVlBcmvjlKusq2-xA7dhK5BUN82BN7X4$) *for guidelines on how to de-identify and prepare clinical data for publication. For a list of recommended repositories, please see* [*https://journals.plos.org/plosone/s/recommended-repositories*](https://urldefense.com/v3/__https:/journals.plos.org/plosone/s/recommended-repositories__;!!Dq0X2DkFhyF93HkjWTBQKhk!SPYwApMAsT9eLGNH3w_aiAr7TwkZW6Ki_ojGrNod2TtmBwl3kc-29D1nIVlBcmvjlKusq2-xA7dhK5BUN3tbfZos$)*. You also have the option of uploading the data as Supporting Information files, but we would recommend depositing data directly to a data repository if possible.
Please update your Data Availability statement in the submission form accordingly.*

Our data availability statement has been updated in the Data availability section and in the submission form.

See Data availability (line 721).

Line 722: “Data cannot be shared publicly because data contain protected health information (including identifying or sensitive patient information). Data cannot be shared publicly even if de-identified due to ethical restrictions from the Institutional Review Board related to risk of reidentification. Data are available from the corresponding author (t-walunas@northwestern.edu) on reasonable request for researchers who meet the criteria for access to confidential data. Note that row-level data are access-controlled and cannot be provided publicly due to data use restrictions”

*5. Please ensure that you refer to Figure 1 in your text as, if accepted, production will need this reference to link the reader to the figure.*

Figure 1 is referred to in the text.

Line 388: “The primary outcome we assessed was in-hospital mortality (Fig 1)”

*6. Please upload a copy of Supporting Information Figure/Table/etc. SI (Fig 1) which you refer to in your text on page 11.*

All supporting information has been uploaded.
