# Supplementary material for: Immunosuppression variably impacts outcomes for patients hospitalized with COVID-19: A retrospective cohort study
Source: PLoS One. 2025 Aug 8;20(8):e0330110. doi: 10.1371/journal.pone.0330110 (PMC12334029; doi:10.1371/journal.pone.0330110)
Supplement: Attachment — Submitted filename: Response_to_Reviewers_auresp_2.docx [file pone.0330110.s010.docx]

Response to Reviewers

Thank you to everyone for their comments on this second round of revisions.

The original comments from the Reviewers are in *italics*, and my responses are below each comment in regular text. When appropriate, line numbers/sections to refer to and specific changes made are available in blue text under each response. Important deletions are marked as “DELETED.” Line numbers refer to those in “Revised Manuscript with Track Changes.”

**Response to Reviewer 1**

*Comment 1: “The article is well structured and well written. It provides a certain contribution to our COVID-19 knowledge.”*

We thank the reviewer for taking the time to review this article.

**Response to Reviewer 2**

*Comment 1: “ICU hospitalization has been clarified. The differences in table 2 and 3 are now clear as explained. The discrepancy in paragraph 4 in the discussion section has been addressed.”*

We are glad these aspects are clear now. We thank the reviewer for taking the time to review this article.

**Response to Reviewer 3**

*Comment 1: “Thank you for taking the time to address all the issues raised in our last round of reviews. Primarily, the capture of discharge disposition allows the researcher to have a first step for determining competing risks. For example, if we are interested in length of stay, then the time to event (TTE) of interest is time to being discharged alive, which makes in-hospital mortality(discharged not alive) a competing risk. Both of these variables should be available in the discharge disposition, in general. Further, one is not burdened with validating whether the Time to event follows a normal distribution or not. More importantly, most of the endpoints of interest in this study are a variation of length of stay, which may by definition, i.e. duration from admission to discharge, focuses on the interval and ignores status of patient at discharge. So time to in-hospital mortality recognizes that intention to capture duration to the occurrence of death. As long as a patient admitted to the hospital or the ICU, and the possibility of death during the stay exists then for those that are not discharged alive, then the ability for evaluating the true LOS may have been terminated, I appreciate that this will be cited as a limitation, but it has not been explained which status of the patient at discharge can not be utilized to sort between the two types of length of stay, so that the researcher/reader is aware of how much bias of LOS will be present in the results of the current study, and showing the extent to which the findings are still valuable.”*

We appreciate the reviewer for emphasizing the importance of stratifying the length outcomes by discharge disposition to provide more granular information about the true lengths of stay for these patients (i.e., differentiating between time to in-hospital mortality and time to being discharged alive). Based on this feedback, we have performed an additional analysis of hospitalization length and ICU length of stay sorted by those who were eventually discharged and those who died before discharge, as suggested. This new analysis is discussed in the text (in the methods, results, and discussion section), and the full results are available in S4 Table. This will help the researcher/reader better interpret length of stay from this study and strengthen this article, and we thank the reviewer for their feedback.

Line 230: “For both hospitalization length and ICU length, stratified analyses of time to either discharge alive or time to in-hospital mortality were also performed to account for competing risks.”

Line 381: “This remained true when stratifying hospitalization length into length to discharge alive and length to in-hospital mortality.”

Line 385: “However, when stratifying by discharge disposition, only ICU length of stay to in-hospital mortality was significantly longer.”

Line 390: “However, there was no significant difference when we stratified by discharge disposition.”

Line 498: “For the hospitalization length and ICU length of stay outcomes, we accounted for competing risks by performing additional analyses stratifying by discharge disposition.”

See S4 Table (line 709).
