# Supplementary material for: Immunosuppression variably impacts outcomes for patients hospitalized with COVID-19: A retrospective cohort study
Source: PLoS One. 2025 Aug 8;20(8):e0330110. doi: 10.1371/journal.pone.0330110 (PMC12334029; doi:10.1371/journal.pone.0330110)
Supplement: Attachment — Submitted filename: Response to Reviewers.20250701.docx [file pone.0330110.s011.docx]

**Response to Reviewer 3**

*Thank you for your time responding to comments previously raised on competing risks.*

*There is a methodology for handling competing risks. The recent review includes the strategy of presenting the analysis into two strata: those that die during the hospital stay and those that were discharged alive. The methodology evaluates cumulative incidence using cumulative incidence function (CIF) as opposed to usual KM curves which can not handle competing risks; and comparison of the CIF is conducted using Gray's test, instead the classing log-rank test. Since this methods accounts competing risks, the results of primary outcome of interests are presented on the entire data and not by stratum, which does not resolve the bias introduced by ignoring the competing risk structure of the occurrence of the events in this problem. Please consider employing the unified approach for handling TTE in the presence of competing risk; i.e. death during hospitalization.*

We thank the reviewer for the additional feedback. As requested, we employed a unified approach for handling TTE using competing risks methodology. Discharge alive was treated as the primary event, with death during hospitalization as a competing risk. Analyses included cumulative incidence functions (CIFs) for discharge, Gray’s tests to compare discharge CIFs across exposure subgroups, and Fine-Gray proportional sub-distribution hazards models to quantify subgroup associations. This approach aligns with modern standards for time-to-event data with competing events.

Methods: “To account for competing risks when comparing hospitalization length and ICU length of stay, time-to-event analyses were also performed with discharge alive as the event of interest and in-hospital death as the competing event. Gray’s test was used to compare the cumulative incidence functions of discharge between exposure subgroups and a Fine-Gray proportional sub-distribution hazards model was implemented to examine the association between exposure subgroup and discharge incidence adjusting for the same covariates.”

Results: “To account for the competing risk of in-hospital mortality on hospitalization length and ICU length of stay, we also compared the cumulative incidence functions of discharge between exposure groups using Gray’s test and performed Fine-Gray regression analyses of these outcomes. Gray’s test showed that the cumulative incidence functions for hospital discharge and ICU discharge were significantly different between groups (p < 0.001 for hospital discharge, p = 0.013 for ICU discharge). When accounting for competing risks, patients with immunosuppression still had longer time to hospital discharge (adjusted HR: 0.74, 95% CI: 0.69-0.79). This was also true for ICU length of stay (adjusted HR: 0.79, 95% CI: 0.69-0.91). Subgroup analysis still showed the same trends for patients with SOT when accounting for competing risks (adjusted HR hospitalization length: 0.77, 95% CI: 0.70-0.85; adjusted HR ICU length of stay: 0.78, 95% CI: 0.66-0.94). However, PWH with low CD4 also had longer time to hospital discharge when accounting for competing risks (adjusted HR: 0.65, 95% CI: 0.46-0.91). In addition, PWH without low CD4 did not have a significantly different time to ICU discharge when accounting for competing risks (Table 5).”

See Table 5.

Table 5 Legend: “Adjusted hazard ratios are from Fine-Gray regression analyses with age at admission, diabetes, obesity, SARS-CoV-2 variant era, remdesivir use, and Paxlovid use as covariates with in-hospital mortality as the competing risk. Significant hazard ratios are in bold.”

Discussion: “In-hospital mortality, ICU admission, and hospitalization length accounting for competing risks were significantly higher/longer in PWH with low CD4. A cohort study in Italy of PWH with low CD4 cell count hospitalized with COVID-19 also found higher mortality and significantly longer hospitalization in this group compared to the general population. Notably, we did not observe any significant differences in oxygenation requirements or length of ICU stay compared to patients without immunosuppression [25]. It was interesting that we observed a similar trend in mortality and hospitalization length despite other outcomes that were otherwise not worse compared to patients without immunosuppression.”
